# Transportin-2 plays a critical role in nucleocytoplasmic shuttling of oestrogen receptor-α

**DOI:** 10.1038/s41598-020-75631-3

**Published:** 2020-10-29

**Authors:** Tetsuji Moriyama, Yoshihiro Yoneda, Masahiro Oka, Masami Yamada

**Affiliations:** 1 Department of Cell Biology and Biochemistry, Division of Medicine, Faculty of Medical Sciences, University of Fukui, 23-3 Matsuoka Shimoaizuki, Eiheiji-cho, Yoshida-gun, Fukui, 910-1193 Japan; 2 Health and Nutrition (NIBIOHN), National Institutes of Biomedical Innovation, 7-6-8 Saito-Asagi, Ibaraki, Osaka 567-0085 Japan; 3 Laboratory of Nuclear Transport Dynamics, Graduate School of Pharmaceutical Sciences, Osaka University, 1-6 Yamada-oka, Suita, Osaka 565-0871 Japan; 4 Laboratory of Nuclear Transport Dynamics, National Institutes of Biomedical Innovation, Health and Nutrition (NIBIOHN), 7-6-8 Saito-Asagi, Ibaraki, Osaka 567-0085 Japan; 5 Life Science Research Laboratory, University of Fukui, 23-3 Matsuoka Shimoaizuki, Eiheiji-cho, Yoshida-gun, Fukui, 910-1193 Japan

**Keywords:** Protein translocation, Transport receptors

## Abstract

Oestrogen receptor-α (ERα) shuttles continuously between the nucleus and the cytoplasm, and functions as an oestrogen-dependent transcription factor in the nucleus and as an active mediator of signalling pathways, such as phosphatidylinositol 3-kinase (PI3K)/AKT, in the cytoplasm. However, little is known regarding the mechanism of ERα nucleocytoplasmic shuttling. In this study, we found that ERα is transported into the nucleus by importin-α/β1. Furthermore, we found that Transportin-2 (TNPO2) is involved in 17β-oestradiol (E2)-dependent cytoplasmic localisation of ERα. Interestingly, it was found that TNPO2 does not mediate nuclear export, but rather is involved in the cytoplasmic retention of ERα via the proline/tyrosine (PY) motifs. Moreover, we found that TNPO2 competitively binds to the basic nuclear localisation signal (NLS) of ERα with importin-α to inhibit importin-α/β-dependent ERα nuclear import. Finally, we confirmed that TNPO2 knockdown enhances the nuclear localisation of wild-type ERα and reduces PI3K/AKT phosphorylation in the presence of E2. These results reveal that TNPO2 regulates nucleocytoplasmic shuttling and cytoplasmic retention of ERα, so that ERα has precise functions depending on the stimulation.

## Introduction

Oestrogen receptor-α (ERα) plays important roles in various physiological processes, such as male and female reproductive development, lipid and bone metabolism, and the maintenance of the cardiovascular and nervous systems^1, 2^. ERα proteins are primarily located in the nucleus, regardless of ligand binding, but shuttle constantly between the nucleus and the cytoplasm. Upon oestrogen binding, ERα changes conformation that enables dimer formation and active transcription of target genes. Oestrogen-bound ERα has been reported to decrease shuttling and further accumulate in the nucleus^3,4^. Outside the nucleus, a small population of ERα is localised to the plasma membrane^5^, which requires palmitoylation, in interaction with the membrane protein caveolin-1^6^. Oestrogen-bound ERα on the plasma membrane can rapidly induce the mobilisation of intracellular calcium^7^ and activation of PI3K/AKT and MAPK/ERK pathways^8–11^. Abnormal phenotypes of nuclear-only ERα and membrane-only ERα transgenic mice suggested that normal organ development and function require convergence and crosstalk of the classical nuclear and extranuclear receptor pathways^12–17^. Moreover, the cytoplasmic ERα mutant, which forcibly changed the localisation of ERα to the cytoplasm, lost oestrogen-induced transcriptional responses, but still maintained MAPK/ERK activation^18^. Recently, cytoplasmic ERα mutant mice have been generated, and preliminary observations suggest that their phenotypes are similar to those of ERα deficient mice^19^. Therefore, nucleocytoplasmic shuttling plays an important role in the regulation of ERα signalling pathways.

Nucleocytoplasmic transport of macromolecules occurs through the nuclear pore complex (NPC), and is generally dependent on the presence of a specific signal sequence, called nuclear localisation signals (NLSs) and nuclear export signals (NESs). Many nucleocytoplasmic transport pathways are mediated by the importin-β superfamily, which is composed of more than 20 family members in humans, and is functionally classified as importins and exportins. The importin-β superfamily exhibits cargo specificity, but has the common features that recognise the NLS or NES sequences of cargo molecules and the GTP-bound form of Ran (RanGTP), a member of the Ras superfamily, and mediate transport between the nucleus and the cytoplasm^20^. Additionally, seven members of the importin-α family, which function as an adaptor between the NLS-containing cargo molecule and importin-β1, have been identified in humans^21^. Importin-α and importin-β families have been reported to be involved in the nucleocytoplasmic shuttling of steroid hormone receptors. For instance, importin-α/β1 heterodimers, importin-7, importin-8, and importin-13 mediate nuclear import of glucocorticoid receptor (GR)^22–24^. Importin-α/β1 heterodimers also mediate the nuclear import of mineralocorticoid receptor^25^ and androgen receptor (AR)^26^. Moreover, CRM1 (exportin-1)^27^ and exportin-5^28^ can stimulate AR export. The distribution of thyroid hormone receptors is regulated by CRM1^29^, exportin-4, exportin-5, and exportin-7^30^. However, only a few studies have shown the nuclear export of ERα^3,31,32^; thus, the regulatory mechanism in ERα nucleocytoplasmic shuttling remains poorly understood.

Here, we sought to gain possible insights into the mechanism of ERα nucleocytoplasmic shuttling. We found that ERα is transported into the nucleus via two different pathways: active transport by importin-α/β1 and passive diffusion. Our results also show that Transportin-2 (TNPO2, importin-3) enhances the cytoplasmic localisation of ERα, not as a nuclear export receptor, but rather as a cytoplasmic retention factor, while it also competitively inhibits the binding of importin-α. Furthermore, we confirmed that TNPO2 knockdown increased the nuclear localisation of endogenous ERα and suppressed PI3K/AKT activation in the presence of its ligand, 17β-oestradiol (E2). We propose that TNPO2 plays a critical role in determining the distribution of ERα between the nucleus and the cytoplasm in response to stimulation.

## Results

### Oestrogen receptor-α enters the nucleus by two pathways: active transport by importin-α/β1 and passive diffusion

**Figure 1:**
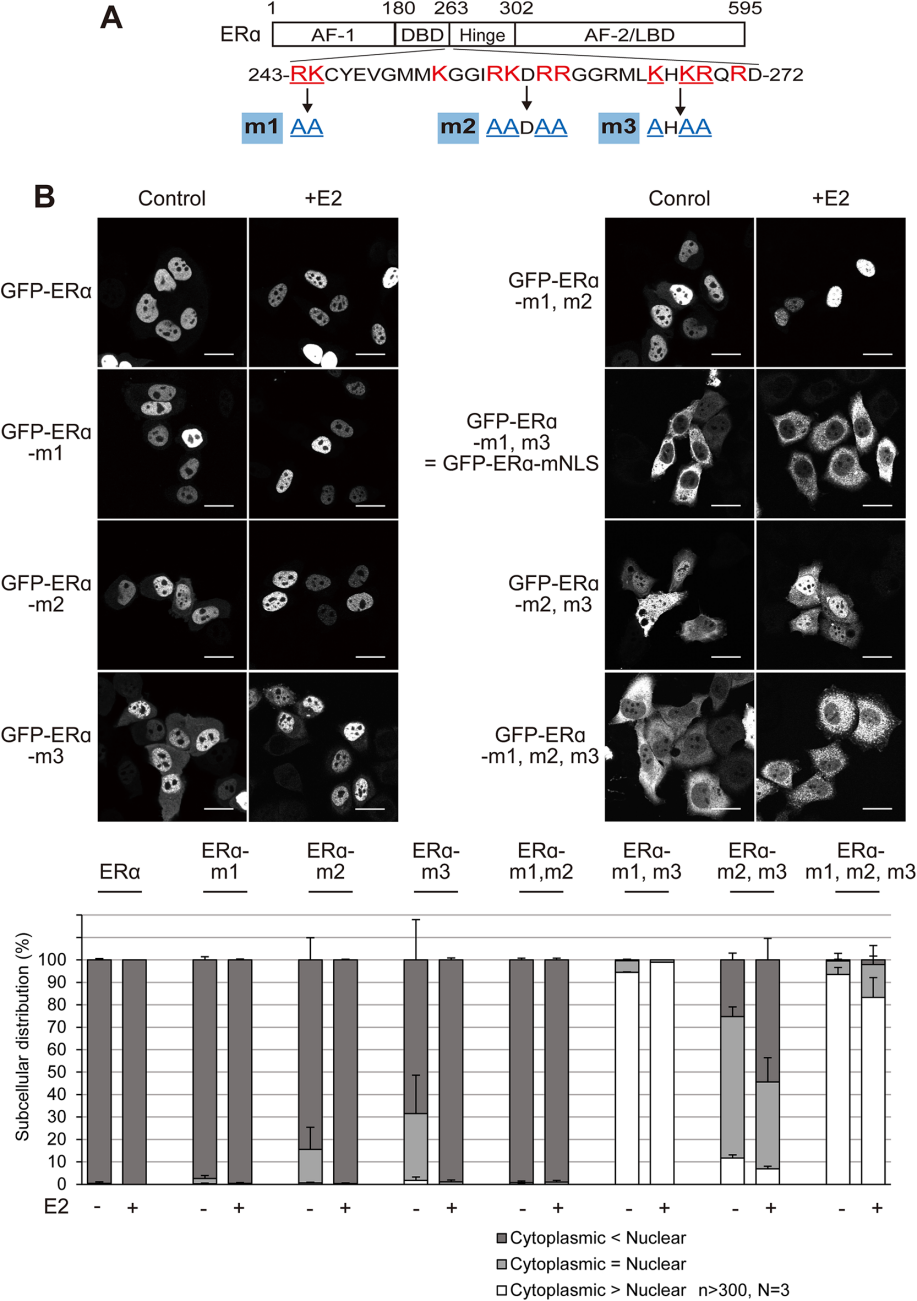
The subcellular distribution of GFP-ERα and the point mutants. (**A**) Schematic representation of the ERα mutants. The diagram shows the full-length ERα, and the positions of the DNA-binding domain (DBD), hinge region, ligand-binding domain (LBD), and the weak constitutive activation function (AF-1), and hormone-dependent activation function (AF-2) are indicated. The NLS straddles the DBD and hinge region. (**B**) Fluorescence images of GFP-ERα and the point mutants in HeLa cells, in the presence or absence of 10 nM E2 for 3 h. Scale bars, 20 µm. The subcellular distribution (%) of GFP-ERα and the point mutants was determined from the nuclear, nuclear and cytoplasmic, and cytoplasmic distribution, calculated from > 300 transfected cells. Each data point represents the average of three independent experiments and the error bars denote the standard deviation.

**Figure 2:**
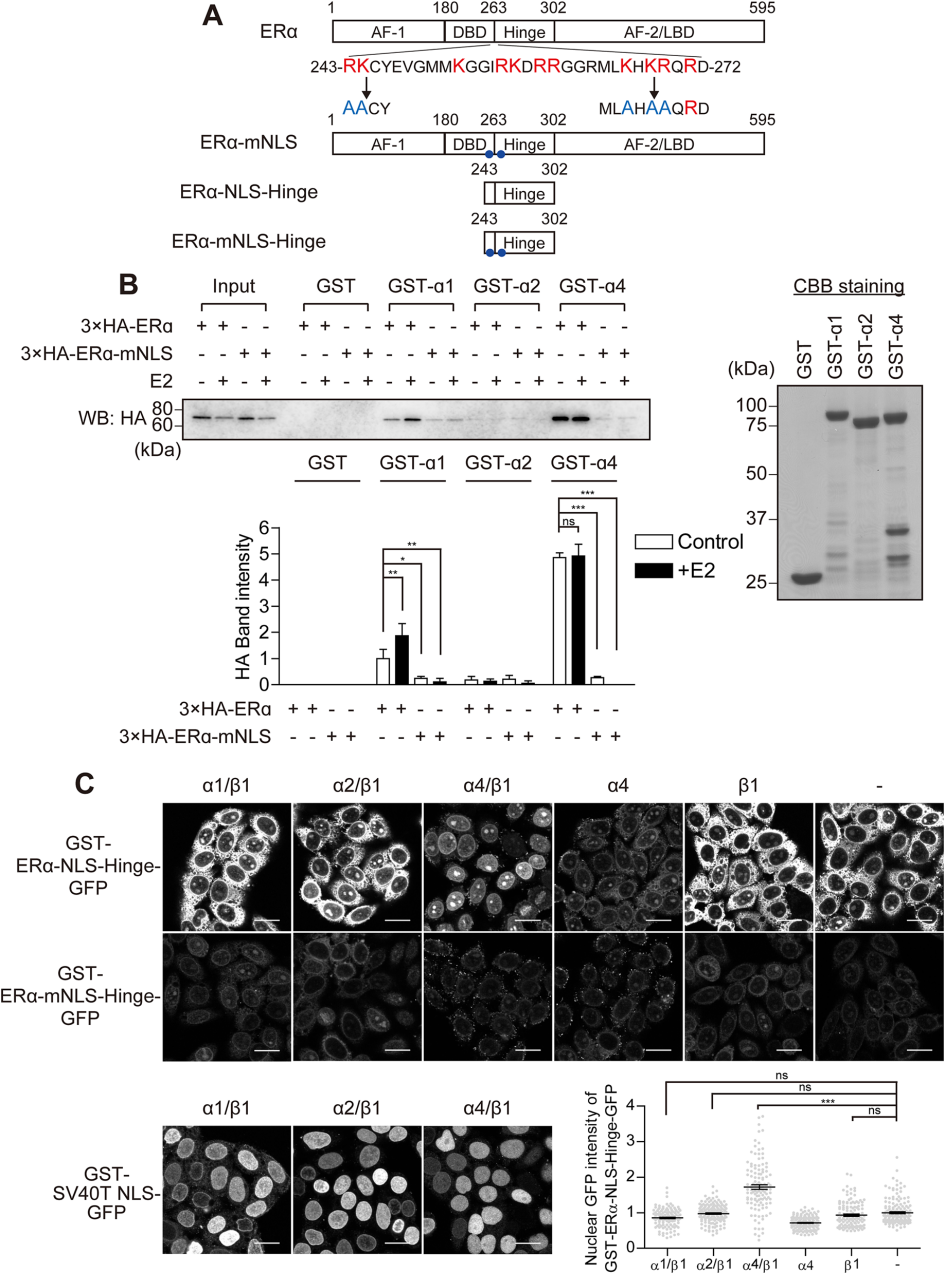
The NLS of ERα is recognised by importin-α/β1. (**A**) Schematic representation of the ERα-NLS mutant (mNLS), ERα-NLS-Hinge and ERα-mNLS-Hinge. (**B**) Importin-α recognises the basic amino acids of ERα. GST pulldown assays were performed with recombinant GST, GST-importin-α1 (GST-α1), GST-importin-α2 (GST-α2) and GST-importin-α4 (GST-α4), using HeLa lysates expressing 3 × HA-ERα or 3 × HA-ERα-mNLS, in the presence or absence of 10 nM E2. CBB stain was used as the loading control for the reaction. The average band intensity of 3 × HA-ERα bound to GST-importin-α1 in the absence of E2 was set to 1. Each data point represents the average of four independent experiments and the error bars denote the standard deviation. *P < 0.05, **P < 0.01, ***P < 0.001; ns, not significant; as determined by one-way ANOVA followed by Tukey’s multiple comparison test. Full-length images of western blot and CBB staining are shown in Supplementary Fig. 9. (**C**) An in vitro transport assay was performed for measuring the nuclear import of GFP-conjugated GST-ERα-NLS-Hinge or GFP-conjugated GST-ERα-mNLS-Hinge, with or without importin-α or importin-β1. The assay was performed in the presence of RanGDP and an ATP-regenerating system containing GTP. GST-SV40T NLS-GFP was used as the positive control. Scale bars, 20 µm. The graphs depict the intranuclear GFP fluorescence intensity of GST-ERα-NLS-Hinge-GFP calculated from the average of at least 120 cells and the error bars denote the respective standard error. ***P < 0.001; ns, not significant; as determined by Kruskal–Wallis test and Dunn’s multiple comparison test. The average nuclear intensity without importin-α and importin-β1 was set to 1.

ERα is predominantly localised in the nucleus, regardless of oestrogen. An NLS of ERα has been located within the DNA-binding domain (DBD) and the hinge region^33,34^. To determine the essential amino acid residues for ERα active nuclear transport, we introduced amino acid substitution mutations in the NLS region by converting the three basic amino acid clusters into alanine (Fig. 1A). Since the molecular weight of ERα is ~ 66 kDa, which could pass the NPC by passive diffusion^35^, we first used ERα mutants with N-terminally fused GFP (~ 103 kDa) (Fig. 1B). When their subcellular distribution was determined in ERα-negative HeLa cells after transient transfection, we observed a dominant nuclear localisation of wild-type ERα (GFP-ERα). Slightly more GFP-tagged mutants of each cluster were found in the cytoplasm as compared with the wild type; however, they were mainly localised in the nucleus, which was further enhanced in the presence of E2. When ERα mutations were combined, GFP-ERα-m1, m2 were also predominantly localised in the nucleus, whereas GFP-ERα-m2, m3 showed a nuclear localisation disruption in the absence of E2; however, this mutant exhibited an increased proportion of nuclear localisation in the presence of E2. In contrast, GFP-ERα-m1, m3 and GFP-ERα-m1, m2, m3 were predominantly localised in the cytoplasm, regardless of the binding of E2. These data indicate that the basic amino acid clusters of both terminals of the NLS region of ERα are necessary and sufficient for ERα nuclear localisation. Therefore, we named ERα-m1, m3 as ERα-mNLS, a mutant defective in active nuclear transport (Fig. 2A). These basic amino acids of the NLS region are potentially recognised by importin-α as a classical NLS (cNLS), linking it to importin-β1 to form the nuclear import complex. Therefore, we examined whether the basic amino acid clusters of ERα are recognised by importin-α1 (KPNA1), importin-α2 (KPNA2), importin-α4 (KPNA4) (Fig. 2B), or importin-β1 (Supplementary Fig. 1) using a glutathione-S-transferase (GST) pulldown assay. In the absence of E2, whole cell lysates prepared from HeLa cells expressing a triple haemagglutinin (3 × HA)-ERα or 3 × HA-ERα-mNLS were incubated with recombinant GST-proteins. 3 × HA-ERα interacted with GST-importin-α4, but 3 × HA-ERα-mNLS mostly did not. In contrast, 3 × HA-ERα and 3 × HA-ERα-mNLS were weakly or not at all bound to GST-importin-α1 and GST-importin-α2. To investigate whether importin-α/β1 mediates the nuclear import of ERα, we subjected the GFP-tagged ERα amino acids 243–302 (GST-ERα-NLS-Hinge-GFP, Fig. 2A) to an in vitro transport assay using digitonin-permeabilized HeLa cells (Fig. 2C). When importin-α4, importin-β1, a small GTPase Ran, and ATP-regeneration system were exogenously supplied, GST-ERα-NLS-Hinge-GFP was efficiently transported into the nucleus, whereas GST-ERα-mNLS-Hinge-GFP was not transported. Nuclear transport by importin-α4 did not appear in the absence of importin-β1, Ran, or the ATP-regeneration system (Supplementary Fig. 2). In contrast, in the case of other importin-αs and in the absence of importin-α4, GST-ERα-NLS-Hinge-GFP fluorescent signal was mainly located in the cytoplasm, whereas the nuclear GFP intensity was equal to that of the negative control (the condition without importin-α nor importin-β1), in accordance with the weak binding of GST-importin-α1 and GST-importin-α2 to 3 × HA-ERα. Moreover, cytoplasmic fluorescence could be suppressed by adding importin-α4. On the other hand, GST-ERα-mNLS-Hinge-GFP showed faint cytoplasmic fluorescence, regardless of importin-α or importin-β1 (see “Discussion” below). Together, these results indicate that importin-α4 recognises ERα through its basic amino acid clusters of the NLS region to form an ERα/importin-α4/β1 complex, and actively transports ERα into the nucleus in a Ran- and ATP-dependent manner. E2 treatment increased the binding of 3 × HA-ERα to GST-importin-α1 (Fig. 2B), although there was almost no change in the binding between GST-importin-α4 and 3 × HA-ERα. Moreover, E2 treatment also enhanced the binding of 3 × HA-ERα to GST-importin-β1 (Supplementary Fig. 1). These data suggest that with E2 treatment, the importin-α/β1 heterodimer may facilitate ERα nuclear transport (see “Discussion” below).

**Figure 3:**
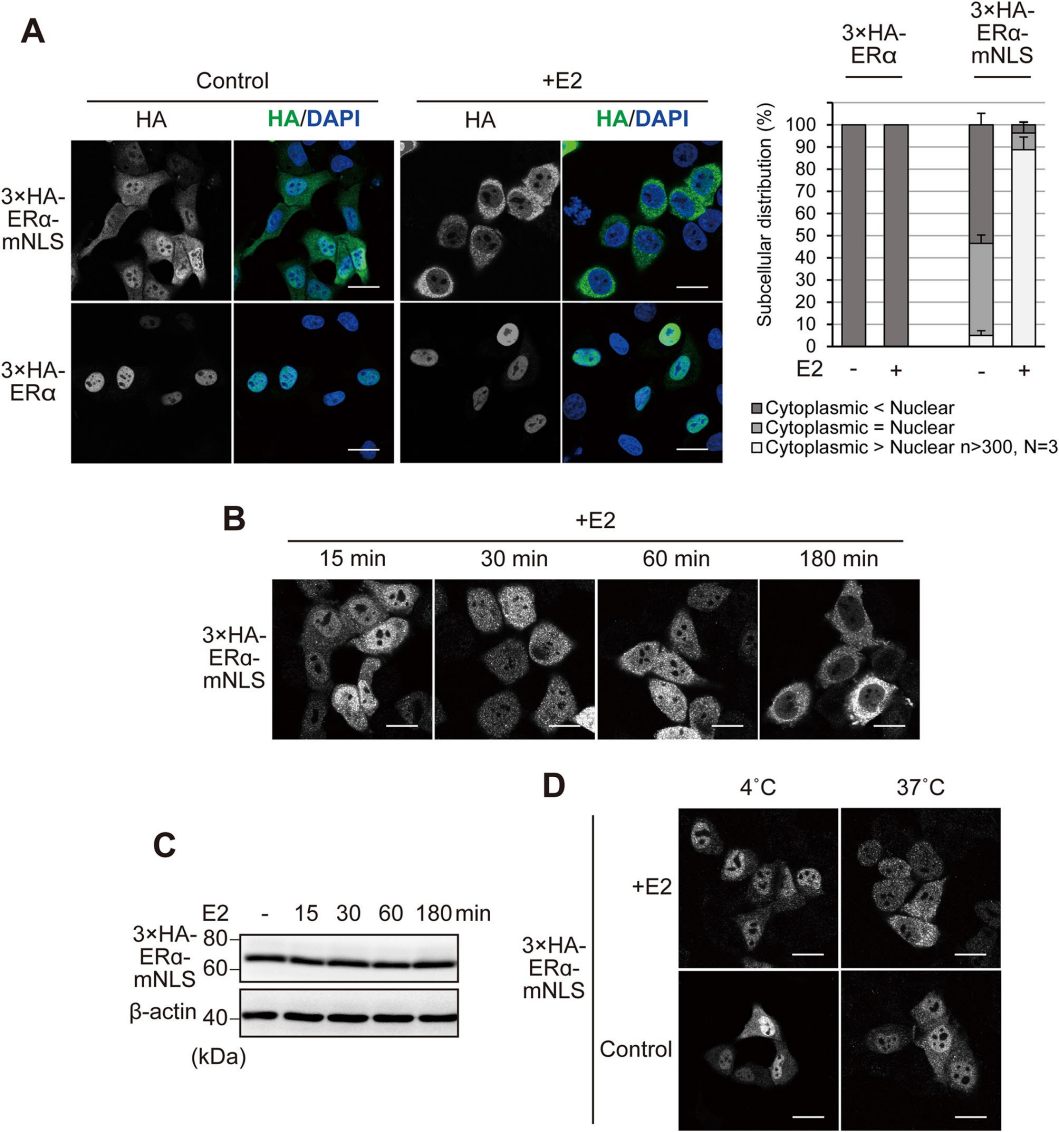
E2 induces cytoplasmic localisation of ERα-mNLS. (**A**) Immunofluorescence images of 3 × HA-ERα and 3 × HA-ERα-mNLS in HeLa cells, in the presence or absence of 10 nM E2 for 3 h. The cells were immunostained for HA (green) and stained with DAPI (blue). Scale bars, 20 µm. The subcellular distribution (%) of GFP-ERα and the point mutants was determined from the nuclear, nuclear and cytoplasmic, and cytoplasmic distribution, calculated from > 300 transfected cells. Each data point represents the average of three independent experiments and the error bars denote the standard deviation. (**B**) Immunofluorescence images of 3 × HA-ERα-mNLS in HeLa cells treated with 10 nM E2 for the indicated durations. The cells were immunostained for HA. Scale bars, 20 µm. (**C**) Treatment with E2 did not affect the steady-state expression levels of 3 × HA-ERα-mNLS. HeLa cells expressing 3 × HA-ERα-mNLS were treated with 10 nM E2 for the indicated durations. Following treatment, the cells were lysed and subjected to SDS-PAGE. Western blotting was performed with an anti-HA antibody. β-actin was used as the loading control. Full-length images of western blots are shown in Supplementary Fig. 10. (**D**) The nuclear export of 3 × HA-ERα-mNLS was inhibited at a lower temperature. HeLa cells expressing 3 × HA-ERα-mNLS were treated with 10 nM E2 and incubated at 4 °C or 37 °C for 1 h and were immunostained for HA. Scale bars, 20 µm.

Next, we analysed the subcellular distribution of 3 × HA-ERα (~ 70 kDa), which is smaller than GFP-ERα, in HeLa cells. 3 × HA-ERα was predominantly localised in the nucleus, corresponding to the distribution of GFP-ERα (Figs. 3A, 1B). On the other hand, 3 × HA-ERα-mNLS was relatively abundant in the nucleus, which is obviously different from the distribution of GFP-ERα-mNLS (Figs. 3A, 1B), probably because of the size of the tag: GFP-ERα, but not 3 × HA-ERα, was too large to passively diffuse through the NPC. These results suggest that endogenous ERα can both actively shuttle and passively diffuse between the nucleus and the cytoplasm.

### E2 induces an ERα-NLS mutant to translocate into the cytoplasm

We investigated the effect of E2 treatment on the subcellular distribution of ERα. 3 × HA-ERα showed only a slight change, but 3 × HA-ERα-mNLS was dramatically translocated to the cytoplasm by E2 treatment (Fig. 3A). The alteration of the distribution pattern started within 30 min after E2 treatment and was increasingly prevalent in the cytoplasm as the time course progressed (Fig. 3B). To exclude the possibility of enhanced degradation of 3 × HA-ERα-mNLS caused by E2 treatment, 3 × HA-ERα-mNLS protein levels of unstimulated and E2-stimulated cells were compared between the 15 and 180 min time points. E2 had no visible effect on the 3 × HA-ERα-mNLS protein levels (Fig. 3C).

Next, we tested whether 3 × HA-ERα-mNLS nuclear export occurs in a passive diffusion process. Incubation of intact cells at a low temperature allows for passive diffusion through the NPC^36,37^. HeLa cells transfected with 3 × HA-ERα-mNLS were incubated at 4 °C or 37 °C with E2 for 1 h. As shown in Fig. 3D, 3 × HA-ERα-mNLS nuclear export was inhibited at low temperature, suggesting that 3 × HA-ERα-mNLS is exported into the cytoplasm via a temperature-sensitive transport mechanism, rather than by passive diffusion.

Previous reports showed that CRM1, which is a ubiquitous nuclear export receptor, mediates ERα nuclear export^3,31^. To examine whether the E2-dependent cytoplasmic localisation of 3 × HA-ERα-mNLS is mediated by CRM1, HeLa cells were transfected with 3 × HA-ERα-mNLS and treated with leptomycin B (LMB), an inhibitor of CRM1-dependent nuclear export^38^. As shown in Supplementary Fig. 3A, LMB did not prevent the alteration of the distribution pattern of ERα-mNLS in the presence of E2, whereas it inhibited the nuclear export of endogenous RanBP1 as previously reported^36^. These contrasting results on the LMB effect in the subcellular localisation of ERα are probably caused by the NLS mutant used in this study instead of the wild type ERα used in the previous reports. Therefore, it is possible that the imbalance in ERα-mNLS transport allowed us to observe the nuclear export more clearly. Meanwhile, we confirmed the binding of recombinant GST-CRM1 and 3 × HA-ERα-mNLS exogenously expressed in HeLa cells using a GST pulldown assay, as described previously^32^ (Supplementary Fig. 3B). Hence, we could not exclude the possibility of ERα nuclear export in a CRM1-dependent manner.

### Transportin-2 is required for the E2-induced cytoplasmic localisation of the ERα-NLS mutant, but does not function as its nuclear export receptor

**Figure 4:**
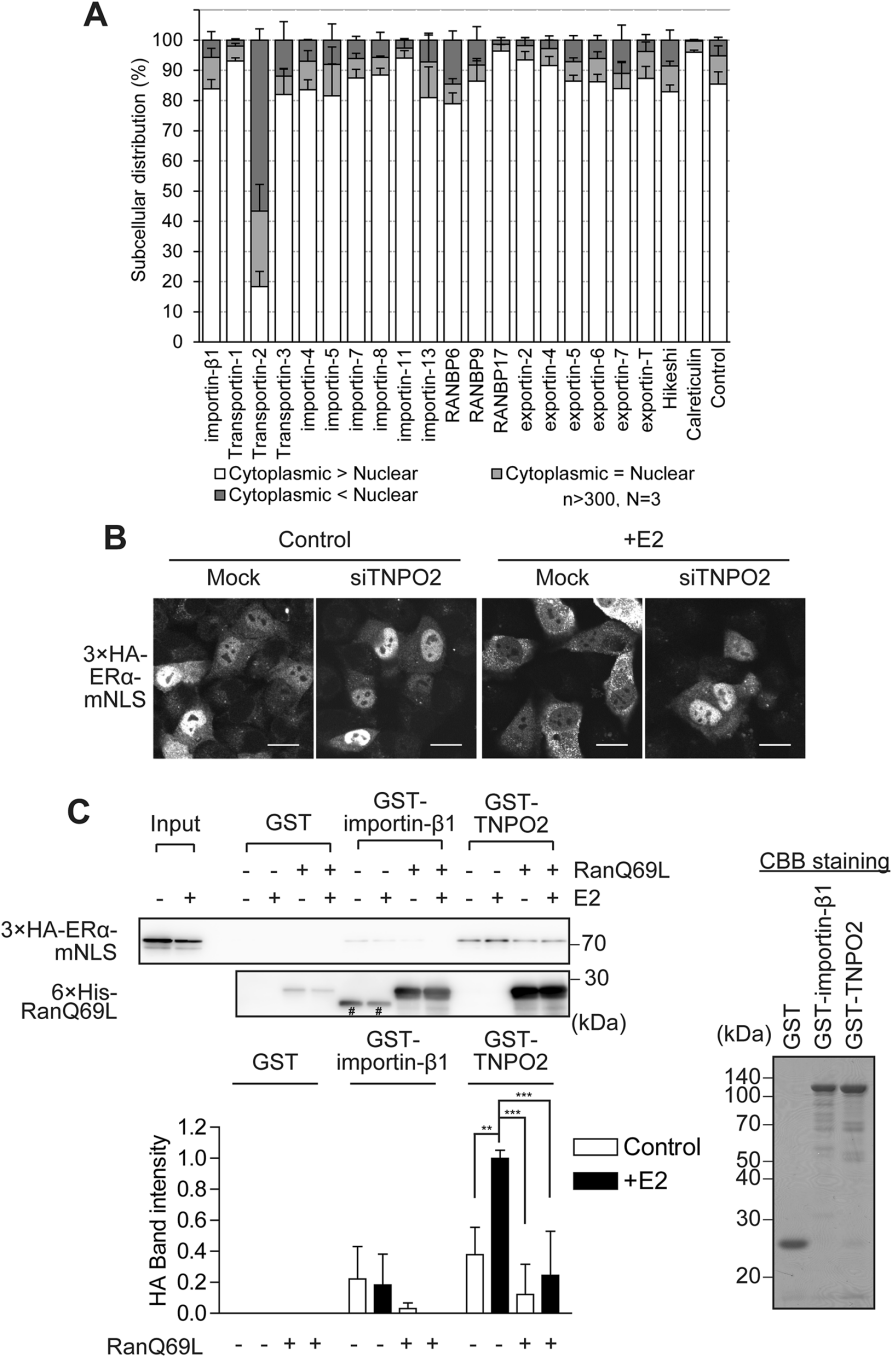
TNPO2 is necessary for the E2-induced cytoplasmic localisation of ERα-mNLS, but does not function as a nuclear export receptor. (**A**) The effect of knockdown of the importin-β family, calreticulin, or hikeshi on the subcellular distribution of 3 × HA-ERα-mNLS. HeLa cells were transfected with siRNAs against the importin-β family, hikeshi, and calreticulin. The next day, the cells were transfected with plasmids expressing 3 × HA-ERα-mNLS. The cells were subsequently treated with 10 nM E2 for 2 h and immunostained for HA. The subcellular distribution (%) of 3 × HA-ERα-mNLS was determined from the nuclear, nuclear and cytoplasmic, and cytoplasmic distribution, calculated from > 300 transfected cells. Each data point represents the average of results obtained from three independent experiments and the error bars denote the standard deviation. (**B**) Immunofluorescence images of 3 × HA-ERα-mNLS in HeLa cells transfected with TNPO2-specific siRNAs, in the presence or absence of 10 nM E2 for 2 h. The cells were immunostained for HA. Scale bars, 20 µm. (**C**) TNPO2 recognises 3 × HA-ERα-mNLS in an E2-dependent and RanGTP-sensitive manner. GST pulldown assays were performed with recombinant GST, GST-importin-β1 and GST-TNPO2, using HeLa lysates expressing 3 × HA-ERα-mNLS, in the presence or absence of 2 µM recombinant 6 × His-RanQ69L. GST-importin-β1 was used as the negative control. #, non-specific band. CBB stain was used as the loading control for the reaction. The average band intensity of 3 × HA-ERα-mNLS bound to GST-TNPO2 in the presence of E2 was set to 1. Each data point represents the average of four independent experiments and the error bars denote the standard deviation. **P < 0.01, ***P < 0.001 as determined by one-way ANOVA followed by Tukey’s multiple comparison test. Full-length images of western blots and CBB staining are shown in Supplementary Fig. 11.

We speculated that the importin-β superfamily mediated ERα export. Therefore, to further study the E2-dependent ERα export mechanism, we investigated the effect of siRNA against importin-β family genes, calreticulin^39,40^ and hikeshi (Hsp70s import receptor)^41^ genes using 3 × HA-ERα-mNLS, as a useful tool to easily observe ERα export. The knockdown efficiency of the corresponding siRNAs was determined using quantitative real-time PCR analysis (Supplementary Fig. 4A) and western blotting for some of the importin-β family members (Supplementary Fig. 4B). After E2 treatment, we examined the subcellular distribution of 3 × HA-ERα-mNLS in siRNA-transfected HeLa cells using immunofluorescence. We found that Transportin-2 (TNPO2, importin-3) siRNA-knockdown led to a strong inhibition of the E2-induced cytoplasmic localisation of 3 × HA-ERα-mNLS, whereas the other genes did not significantly affect its location (Fig. 4A,B). Next, to validate the binding between TNPO2 and ERα, we performed recombinant GST-TNPO2 pulldowns from E2-treated or untreated HeLa cells expressing 3 × HA-ERα-mNLS. In the absence of E2, 3 × HA-ERα-mNLS only slightly interacted with GST-TNPO2, but this interaction was strongly increased by E2 treatment (Fig. 4C). The binding of exportins to cargo molecules is greatly enhanced by RanGTP^42–44^. However, 3 × HA-ERα-mNLS unexpectedly failed to bind to GST-TNPO2 in the presence of the GTPase-deficient mutant RanQ69L (GTP-form) (Fig. 4C), indicating that TNPO2 does not function as a conventional export receptor for 3 × HA-ERα-mNLS. Furthermore, GFP-ERα-mNLS was largely restricted to the cytoplasm in the absence or presence of E2 (Fig. 1B), implying that TNPO2 is not an ERα import receptor. Together, these results indicate that TNPO2 is required for the E2-induced cytoplasmic localisation of ERα-mNLS but does not mediate its nuclear export.

### Transportin-2 binds to ERα via two PY-motifs in the AF-1 domain and facilitates ERα localisation to the cytoplasm in an E2-dependent manner

**Figure 5:**
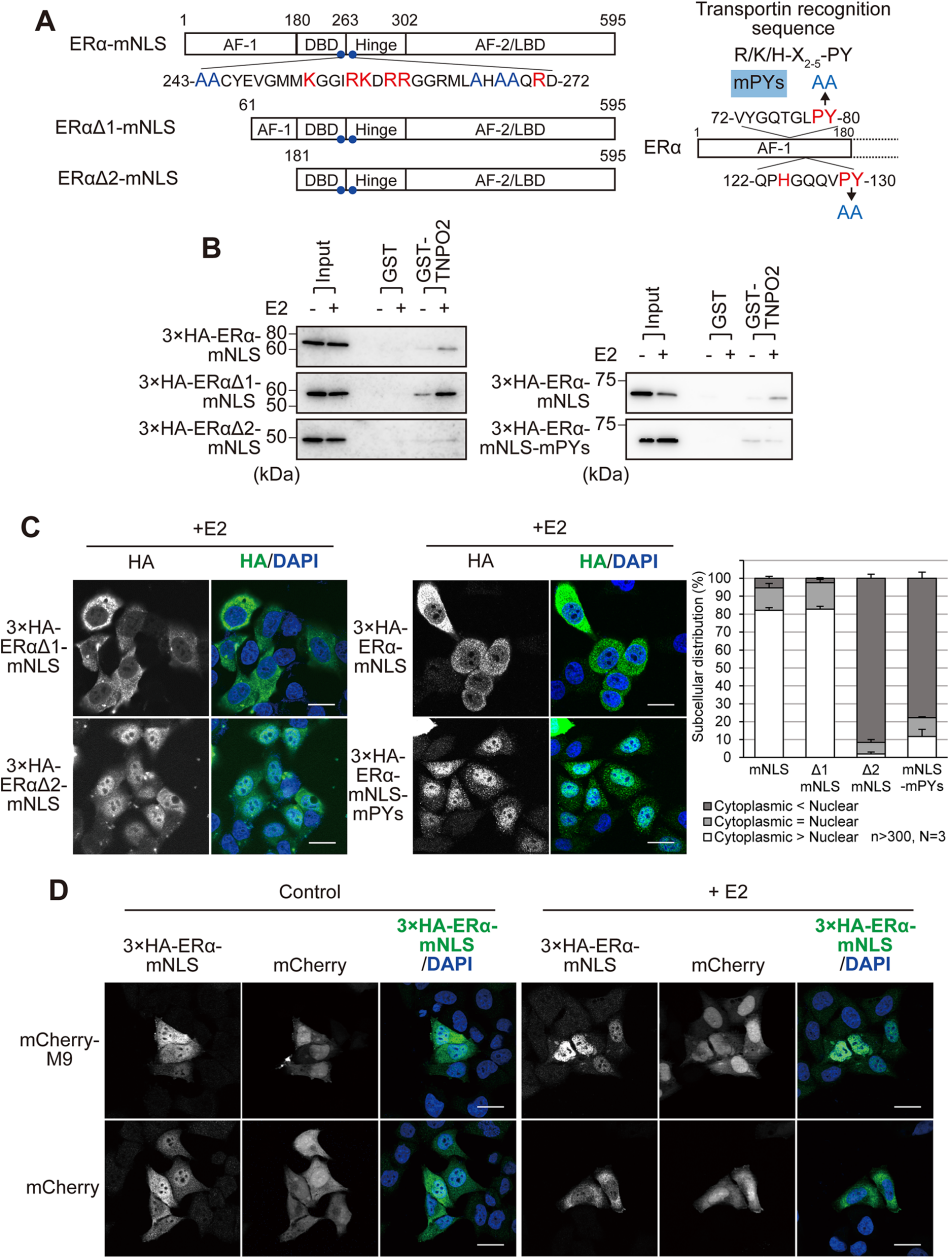
Transportin-2 binds to ERα via two similar PY-motifs in the AF-1 domain. (**A**) Schematic representation of the ERα-mNLS deletion mutants and the ERα-mNLS-mPYs. (**B**) GST pulldown assays were performed with recombinant GST and GST-TNPO2, using HeLa lysates expressing 3 × HA-ERα-mNLS, 3 × HA-ERαΔ1-mNLS, or 3 × HA-ERαΔ2-mNLS (left panel), and 3 × HA-ERα-mNLS or 3 × HA-ERα-mNLS-mPYs (right panel). Full-length images of western blots are shown in Supplementary Fig. 12. (**C**) Immunofluorescence images of 3 × HA-ERαΔ1-mNLS, 3 × HA-ERαΔ2-mNLS, 3 × HA-ERα-mNLS, or 3 × HA-ERα-mNLS-mPYs in HeLa cells, in the presence of 10 nM E2 for 3 h. The cells were immunostained for HA (green) and stained with DAPI (blue). Scale bars, 20 µm. The subcellular distribution (%) of the 3 × HA-ERα-mNLS mutants was determined from their nuclear, nuclear and cytoplasmic, and cytoplasmic distribution, calculated from > 300 HA-positive cells. Each data point represents the average of results obtained from three independent experiments and the error bars denote the standard deviation. (**D**) Immunofluorescence images of HeLa cells co-transfected with 3 × HA-ERα-mNLS and mCherry-M9, in the presence or absence of 10 nM E2, for 2 h. The cells were immunostained for HA (green) and stained with DAPI (blue). Scale bars, 20 µm.

TNPO2 has been reported to recognise consensus sites of cargo molecules containing a characteristic proline/tyrosine (PY) motif^45^, which possesses a central hydrophobic (Φ-G/A/S-ΦΦ, Φ is a hydrophobic residue) or basic motif, followed by a C-terminus R/H/K-X(2–5)-P-Y (X is any residue) consensus sequence^46^. The 61–180 residues of the AF-1 domain of ERα contain two similar PY-motifs: 64-AAAAANAQVYGQTGLPY-80 and 117-LSPFLQPHGQQVPY-130. First, in order to clarify the relationship between ERα and TNPO2, we generated two deletion mutants lacking the AF-1 domain of ERα-mNLS, residues—61–595 (ERαΔ1-mNLS) and 181–595 (ERαΔ2-mNLS), respectively (Fig. 5A, left panel), and examined their binding to GST-TNPO2 and their subcellular distributions. The GST pulldown assay showed that exogenously expressed 3 × HA-ERα-mNLS and 3 × HA-ERαΔ1-mNLS in HeLa cells had higher binding to recombinant GST-TNPO2 after E2 treatment, whereas 3 × HA-ERαΔ2-mNLS showed only little binding capacity (Fig. 5B, left panel). Immunofluorescence staining showed that 3 × HA-ERαΔ1-mNLS and 3 × HA-ERαΔ2-mNLS were relatively abundant in the nucleus in the absence of E2, similar to 3 × HA-ERα-mNLS (Supplementary Fig. 5A). However, after E2 treatment, 3 × HA-ERαΔ1-mNLS was still translocated to the cytoplasm, while 3 × HA-ERαΔ2-mNLS was not (Fig. 5C, left panel). These results indicate that the AF-1 domain of ERα (amino acids 61–180), which includes two putative PY-motifs, is required for E2-dependent binding to TNPO2 and for ERα-mNLS localisation to the cytoplasm. To further clarify this, we constructed a double PY-motifs mutant (mPYs) that replaced the PY-motif (P79/Y80 and P129/Y130) with alanine (Fig. 5A, right panel). As expected, 3 × HA-ERα-mNLS-mPYs exogenously expressed in HeLa cells almost lost the ability to interact with recombinant GST-TNPO2 in the GST pulldown assay (Fig. 5B, right panel). Moreover, it obviously reduced the cytoplasmic localisation in the presence of E2, compared with 3 × HA-ERα-mNLS (Fig. 5C, middle panel). In the absence of E2, 3 × HA-ERα-mNLS-mPYs was relatively abundant in the nucleus, similar to 3 × HA-ERα-mNLS (Supplementary Fig. 5B). Next, we investigated the effect of the M9 transport signal, which is a transportin-specific substrate^47,48^, on the subcellular distribution of 3 × HA-ERα-mNLS. In mCherry-M9 transfected HeLa cells, the cytoplasmic localisation of 3 × HA-ERα-mNLS was suppressed, remaining mainly in the nucleus regardless of E2 (Fig. 5D). These data indicate that TNPO2 facilitates the cytoplasmic localisation of ERα-mNLS by binding to PY-motifs of ERα in an E2-dependent manner.

### Transportin-2 competes with importin-α for binding to the NLS region of ERα

**Figure 6:**
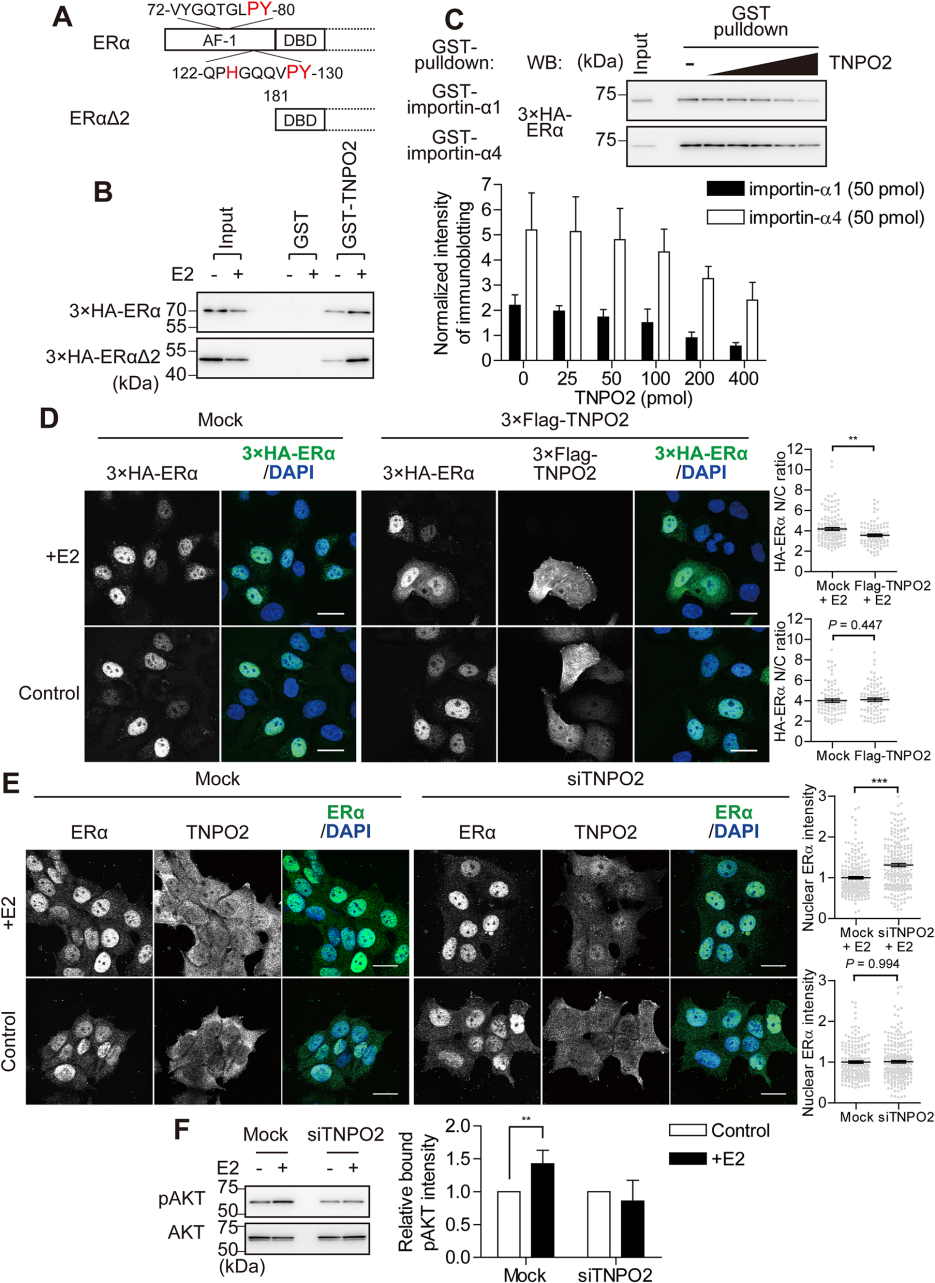
Transportin-2 and importin-α competitively binds to the basic region of the NLS of ERα for modulating its function. (**A**) Schematic representation of the ERα mutants. (**B**) GST pulldown assays were performed with recombinant GST and GST-TNPO2, using HeLa lysates expressing 3 × HA-ERα or 3 × HA-ERαΔ2. (**C**) Competitive binding of importin-α1 or importin-α4 and TNPO2 to ERα. GST-importin-α1 or GST-importin-α4 were immobilised on glutathione Sepharose beads and incubated with HeLa lysates expressing 3 × HA-ERα, in the presence of 10 nM E2. The beads were washed and incubated with different amounts of TNPO2. The bound fraction was analysed by immunoblotting for HA. The quantities of GST-importin-α1, GST-importin-α4, and 3 × HA-ERα complexes are represented graphically. Intensity of 3 × HA-ERα was normalised to the intensity of the input. Each data point represents the average of results obtained from three independent experiments and the error bars denote the standard deviation. (**D**) Immunofluorescence images of HeLa cells co-transfected with 3 × HA-ERα and 3 × Flag-TNPO2, in the presence or absence of 10 nM E2, for 10 min. The cells were immunostained for HA (green) and Flag, and stained with DAPI (blue). Scale bars, 20 µm. The graphs depict Nuclear/Cytoplasm (N/C) ratio of 3 × HA-ERα staining intensity calculated from the average of at least 90 cells and the error bars denote the corresponding standard error. **P < 0.01 determined by the Mann–Whitney test through comparison with the mock samples, in the presence or absence of E2. (**E**) Immunofluorescence images of endogenous ERα and TNPO2 in MCF-7 cells transfected with or without TNPO2-specific siRNAs, in the presence of 10 nM E2, for 10 min. The cells were immunostained for ERα (green) and TNPO2 (red). Scale bars, 20 µm. The graphs depict the intranuclear ERα staining intensity, averaged from at least 180 cells and the error bars denote the average standard errors. ***P < 0.001, the Mann–Whitney test, compared with the mocks, in the presence or absence of E2. (**F**) TNPO2 knockdown inhibited the E2-dependent AKT activation. MCF-7 cells were transfected with TNPO2-specific siRNAs, and treated with 10 nM E2 for 15 min. The cells were lysed and subjected to SDS-PAGE. Western blotting was performed using anti-phosphorylated-AKT and anti-AKT antibodies. The pAKT/AKT ratio in the absence of E2 was set to 1. Each data point represents the average of data from five independent experiments and the error bars denote the standard deviation. **P < 0.01, the Paired ratio t-test, compared with the control. Full-length images of western blots are shown in Supplementary Fig. 13.

Next, we investigated the binding of wild-type ERα and its deletion mutant lacking the AF-1 domain (ERαΔ2) (Fig. 6A) to TNPO2, using a GST pulldown assay. Unexpectedly, both full-length ERα and ERαΔ2, despite not having the two PY-motifs, increased the binding to recombinant GST-TNPO2 after E2 treatment (Fig. 6B). This result suggests that TNPO2 can directly or indirectly bind to the basic NLS region and/or PY-motifs region of ERα in an E2-dependent manner. Since importin-α recognises the NLS region of ERα (Fig. 2B), we examined whether TNPO2 directly completes importin-α off the preformed importin-α-ERα complex. For this purpose, GST-importin-α1 or GST-importin-α4 bound to glutathione Sepharose beads were first incubated with whole cell lysates prepared from E2-treated HeLa cells expressing 3 × HA-ERα protein, and then recombinant TNPO2 was added to the reactions. As shown in Fig. 6C, E2-bound ERα was dissociated from GST-importin-α4 by the addition of TNPO2 in a dose-dependent manner. Similarly, GST-importin-α1 slightly released E2-bound ERα upon TNPO2 addition in a dose-dependent manner. To rule out the possibility that TNPO2 directly binds to importin-α and destabilises the preformed importin-α-ERα complex, we checked for their direct interaction using a GST pulldown assay. We found that recombinant 3 × Flag-importin-α1 and 3 × Flag-importin-α4 did not interact significantly with recombinant GST-TNPO2 (Supplementary Fig. 6). Collectively, these data demonstrate that, in the presence of E2, TNPO2 binds competitively with importin-α to the basic NLS region of ERα.

### Transportin-2 is required for cytoplasmic retention of ERα and concomitant AKT activation

Previous studies demonstrated that, in addition to regulation of the target gene expression, E2-bound ERα can rapidly stimulate the cytoplasmic signalling pathways, including PI3K/AKT^8,11^. Given that TNPO2 could bind to two PY-motifs of ERα in the cytoplasm and competitively bind to importin-α in its basic NLS region in an E2-dependent manner, we speculated that TNPO2 may function in the cytoplasmic retention of ERα to induce cytoplasmic signalling pathways. To further investigate the role of TNPO2 in the regulation of ERα localisation, we co-transfected HeLa cells with 3 × HA-ERα and 3 × Flag-TNPO2 expression vectors. Immunofluorescence staining showed that 3 × HA-ERα was mainly localised in the nucleus of untreated HeLa cells, with or without co-expression of 3 × Flag-TNPO2. However, upon E2 treatment for 10 min, 3 × HA-ERα shifted more to the cytoplasm when the 3 × Flag-TNPO2 was co-expressed, as compared with 3 × HA-ERα only (Fig. 6D). Additionally, overexpression of TNPO2 in ERα-positive MCF-7 cells caused a noticeable change in the subcellular distribution of endogenous ERα from the nucleus to the cytoplasm after E2 treatment for 10 min (Supplementary Fig. 7). Conversely, the suppression of TNPO2 caused an increase in the nuclear endogenous ERα compared with the control cells (Fig. 6E, Supplementary Fig. 8A). These data suggest that TNPO2 functions as a cytoplasmic retention factor for ERα in an E2-dependent manner. Additionally, as shown in Supplementary Fig. 8B, TNPO2 was localised on the plasma membrane and colocalised with ERα in MCF-7 cells, both of which were diminished upon TNPO2 knockdown (see “Discussion” below). To verify the role of TNPO2 in E2-bound ERα-mediated cytoplasmic signalling, we knocked down TNPO2 expression in MCF-7 cells, followed by E2 treatment and assessed the activation of AKT. As shown in Fig. 6F, an increase in AKT phosphorylation was observed in control cells after 15 min of E2 treatment, but not in TNPO2 knockdown cells, indicating that TNPO2 is required for E2-induced activation of AKT.

## Discussion

**Figure 7:**
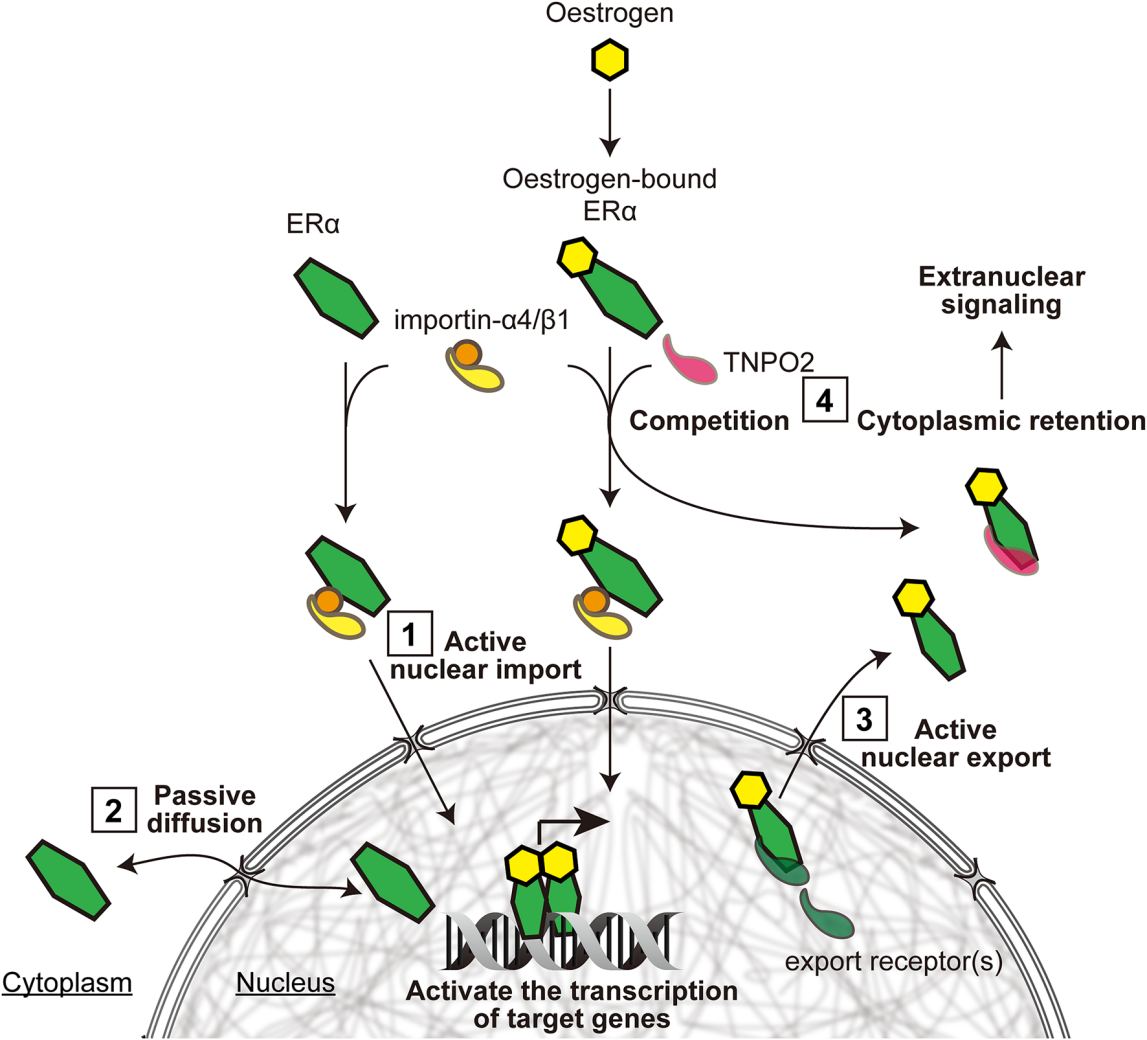
A model for the nucleocytoplasmic shuttling of ERα, regulated by the importin family. Following cytoplasmic translation, ERα translocates into the nucleus by active transport mediated by importin-α4/β (1). ERα can also shuttle between the nucleus and cytoplasm by passive diffusion (2). Simultaneously, nuclear export receptor(s) export ERα from the nucleus (3). In the cytoplasm, TNPO2 shields the NLS from importin-α and increases the cytoplasmic retention of ERα in an E2-dependent manner (4).

Oestrogen receptors belong to the steroid hormone receptor superfamily. The nucleocytoplasmic transport mechanism of steroid hormone receptors has been extensively studied, and many have been reported to involve the importin family^21,23,24,29,46^. However, ERα nuclear transport has still not been clarified. Here, our data provide evidence that ERα is recognised by importin-α4 through its basic amino acid clusters of the NLS region to form an ERα/importin-α4/β1 complex, and can enter the nucleus via active transport. In addition, our observations support the hypothesis that ERα can passively diffuse between the nucleus and the cytoplasm. Furthermore, we have demonstrated that TNPO2 regulates the subcellular distribution of ERα through cytoplasmic retention and competitive inhibition with importin-α in an E2-dependent manner, activating the function of ERα in the cytoplasm as an active mediator of signalling pathways (Fig. 7).

So far, it has been reported that some importins could inhibit the nuclear transport mediated by other importins. For example, importin-α inhibits the nuclear import of Snail mediated by importin-β1^49^. Importin-α2 plays a role in the cytoplasmic retention of Oct6 and Brn2 transcription factors in undifferentiated mouse embryonic stem cells^50^. Importin-7 has also been reported to inhibit the nuclear import of AR by importin-α/β1^51^. We recently found that importin-α8 (KPNA7) forms heterodimers with other importin-α, and proposed that importin-α8 can suppress cNLS binding through hetero-dimerization^52^. Here, we found that TNPO2 could competitively inhibit the nuclear import of ERα by importin-α/β1. The transportin subfamily was first suspected to act as a nuclear bidirectional receptor; however, subsequent studies refuted this hypothesis. It is thought that the transportin subfamily in nucleocytoplasmic transport is restricted to nuclear import^45^. As a typical example, TNPO1 and TNPO2 mediate nuclear import of heterogeneous nuclear ribonucleoprotein (hnRNP) A1 through the interaction of the M9 transport signal. However, even though M9 is known to serve as a bidirectional signal, a nuclear export receptor that recognises M9 is yet to be identified^53,54^. Interestingly, similar to our results, transportin can bind to Deleted in breast cancer 1 (DBC-1) with and without NLS^55^. Therefore, the transportin subfamily may play a role in the regulation of cytoplasmic signalling as well as in nuclear transport.

Of note, our data show that TNPO2 is localised on the plasma membrane and colocalises with ERα in MCF-7 cells, both of which were diminished in TNPO2 knockdown cells (Supplementary Fig. 8B). Therefore, we speculate that TNPO2 may play an important role in ERα translocation to the plasma membrane and promote association with partner proteins, such as IGF-1R^9^, Shc^56^, HER-2^10^, and EGFR^11^. In the future, an accurate understanding of the cytoplasmic roles of the transportin subfamily may provide insight into the novel regulatory mechanism of the biological function of transportin-cargos.

ERα is mainly localised in the nucleus, regardless of the ligand. We have demonstrated that importin-α4 recognises ERα independent of E2 through the basic NLS region. In addition, the importin-α4/β1 heterodimer can effectively import the basic NLS-containing fragment of ERα in an in vitro transport assay, in the presence of Ran and ATP. These data strongly indicate that the importin-α4 subtype constitutively contributes to the nuclear localisation of ERα. Moreover, E2 treatment increased the binding of ERα to importin-β1 (Supplementary Fig. 1), which raises the possibility that E2 may induce the formation of ERα/importin-α4/importin-β1 complex, and promote its nuclear transport. Notably, in the in vitro transport assay, lack of importin-α4 resulted in a pronounced localisation of GST-ERα-NLS-Hinge-GFP to the cytoplasm. Cytoplasmic fluorescence could be suppressed by importin-α4 addition or mutating in the basic amino acids of the NLS. Previous studies have shown that basic proteins aggregate in an importin-free cytosol, apparently through multivalent ionic interactions with cytoplasmic polyanions, such as tRNA^57^. Our results also have led to the idea that importin-α4 may have another function as a cytoplasmic chaperone for the exposed domains with basic charges of ERα.

Oestrogen has been reported to induce nuclear accumulation of ERα^33,34^. Interestingly, the GST pulldown assay showed that E2 treatment increased the binding between ERα and importin-α1 but did not affect the binding between ERα and importin-α4 (Fig. 2B). Similarly, importin-β1 increased the interaction with ERα in response to E2 (Supplementary Fig. 1). These results imply that, in addition to the NLS region, importin-α1 may recognise other region(s) or additional cofactor(s) depending on the conformational change induced by E2 treatment. Thus, the nuclear transport of importin-α1/β1 may be particularly important for the nuclear accumulation of ERα via E2 treatment. Previously, we generated and examined knockout (KO) mice lacking importin-α1^58^. The KO mice showed significantly reduced E2-responsive ERα downstream genes and exhibited not only morphological but also functional deterioration of the female reproductive tract^58^. Importin-α1 deficiency may have caused a decrease in the nuclear accumulation of ERα triggered by oestrogen and affected its transcriptional activity.

The subcellular distribution of 3 × HA-ERα-mNLS dramatically changed to the cytoplasm in the presence of E2 (Fig. 3). Moreover, our data using ERα-mNLS suggest that ERα is exported from the nucleus by (an) other nuclear export receptor(s), in addition to the previously reported CRM1^3,31,32^ (Supplementary Fig. 3), although we could not identify the receptor(s) in this study. Some nuclear hormone receptors have been reported to be exported from the nucleus via more than one pathway^40^. Thus, ERα is likely to have multiple export pathways.

Endocrine therapy is the primary treatment strategy for ERα-positive breast cancer. However, long-term endocrine therapy (such as tamoxifen and fulvestrant) facilitates translocation of ERα to the cytoplasm and repopulation of cancer cells that have acquired resistance to this treatment^59,60^. Clarifying the ERα shuttling mechanism will lay the groundwork for understanding how ERα trafficking changes in breast cancer before/after endocrine therapy, and to create a new method that combines ERα transport mechanisms for effective treatment. In summary, our research has demonstrated that TNPO2 controls ERα nucleocytoplasmic shuttling by cytoplasmic retention and inhibition of importin-α/β1-dependent nuclear import depending on the stimulation, and thus plays a critical role in the proper regulation of the biological functions of ERα.

## Methods

### Plasmid construction

Human ERα, importin-α1, importin-α2, importin-α4, importin-β1 and TNPO2 were generated by polymerase chain reaction (PCR) amplification from total RNA from MCF-7 and HeLa cells using appropriate primers (Supplementary Table ), and subsequently subcloned into the pENTR vector (Thermo Fisher Scientific, Waltham, MA, USA). ERα mutants and importin-α ΔIBB were constructed by site-dir1ected mutagenesis and by inverse PCR mutagenesis with the KOD-Plus-Neo, Ligation high Ver.2, and T4 Polynucleotide Kinase enzymes (KOD-401, LGK-201, PNK-111, TOYOBO, Osaka, Japan) using primers (Supplementary Table 1). Then, using the Gateway system (Thermo Fisher Scientific), the genes were subcloned into p3 × FLAG-CMV DEST^61^, pcDNA3.1/Zeo(+)-EGFP DEST^62^, and pcDNA3.1/Zeo(+) DEST, pGEX-6P-2 DEST, which were generated by a method similar to^62,63^ using pcDNA3.1/Zeo(+) (Thermo Fisher Scientific), pGEX-6P-2 (Cytiva, Piscataway, NJ, USA) and the Reading Frame Cassette of the Gateway Conversion System (Thermo Fisher Scientific). pCold II Ran Q69L was constructed by inserting the human Ran Q69L mutant into pCold II DNA (Takara Bio Inc., Shiga, Japan).

### Cell culture

HeLa and MCF-7 cells were cultured in Dulbecco’s modified Eagle’s medium (D-MEM; 044-29765, FUJIFILM Wako Pure Chemical Corporation, Osaka, Japan) supplemented with 10% foetal bovine serum (FBS). The cells were maintained in a 37 °C incubator with 5% CO2-humidified air.

For E2 treatment, cells were maintained in 5% Charcoal/Dextran-treated FBS (Thermo Fisher Scientific) in phenol red-free D-MEM (044-32955, FUJIFILM Wako Pure Chemical Corporation) for 2 days, and then serum-free media before experimentation.

### In vitro transport assay

An in vitro transport assay was performed as described previously^65,66^. Briefly, HeLa cells were grown on multiwell plates (TF2404; Matsunami Glass Ind., Ltd., Osaka, Japan) for 2 days. After washing with ice-cold transport buffer (TB; 20 mM HEPES [pH 7.3], 110 mM potassium acetate, 2 mM magnesium acetate, 5 mM sodium acetate, 0.5 mM EGTA, 2 mM DTT, 1 μg/mL aprotinin, 1 μg/mL leupeptin, and 1 μg/mL pepstatin), the cells were permeabilised with 20 µg/mL Digitonin, High Purity (300410, Merck Millipore, Burlington, MA, USA) in TB on ice for 5 min. Cells were then washed and immersed in ice-cold TB for 10 min. Preincubated 400 nM GFP-cargo-substrate, 600 nM importin-α, 400 nM importin-β1 and 4 µM RanGDP were mixed with 500 µM GTP and an ATP-regeneration system (3.3 µM ATP, 15 µM phosphocreatine, and 60 U/mL creatine phosphokinase) in TB containing 1% BSA. After applying the mixture, the digitonin-permeabilized cells were incubated at 30 °C for 30 min. Finally, the cells were rinsed with ice-cold TB and fixed with 3.7% formaldehyde in TB for 15 min. The cells were then stained with DAPI.

### Transient transfection

Transfections were performed using Lipofectamine 2000 Transfection Reagent (Thermo Fisher Scientific) and Screen*Fect* A *plus* (FUJIFILM Wako Pure Chemical Corporation) according to the manufacturer's instructions. siRNA duplexes were reverse transfected into HeLa and MCF-7 cells using Lipofectamine RNAiMAX Transfection Reagent (Thermo Fisher Scientific) as recommended by the manufacturer.

### Antibodies

ERα Antibody (F-10) (sc-8002, Santa Cruz Biotechnology, Dallas, TX, USA), Anti-HA-tag mAb (M180-3, MBL, Nagoya, Japan), Monoclonal ANTI-FLAG M2 antibody produced in mouse (F1804, Merck Millipore), Ran BP-1 Antibody (M-45) (sc-28576, Santa Cruz Biotechnology), Actin Antibody (C-11) (sc-1615, Santa Cruz Biotechnology), Purified Mouse Anti-Karyopherin β (610559, BD Transduction Laboratories, San Jose, CA, USA), Purified Mouse anti-Transportin (558660, BD Transduction Laboratories), TNPO2 Polyclonal antibody (17831-1-AP, ProteinTech, Chicago, IL, USA), IPO4 Rabbit pAb (A15600, ABclonal, Cambridge, MA, USA), Anti-Importin 7 antibody (ab99273, abcam, Cambridge, MA, USA), Anti-Cellular Apoptosis Susceptibility/CSE1L antibody (ab96755, abcam), Purified Mouse Anti-Ran (610340, BD Transduction Laboratories), Phospho-Akt (Ser473) (D9E) XP Rabbit mAb (4060, Cell Signaling Technology, Danvers, MA, USA), Akt (pan) (C67E7) Rabbit mAb (4691, Cell Signaling Technology) were used.

### siRNA oligos

Importin-β family, Hikeshi and CALR Silencer Select (Pre-designed) siRNAs were synthesized by Thermo Fisher Scientific: importin-β1 (ID: s7917), TNPO1 (ID:s7933), TNPO2 (ID:s26881), TNPO3 (ID:s24032), importin-4 ID:s36153), importin-5 (ID:s7935), importin-7 (ID:s20640), importin-8 (ID:s20637), importin-11 (ID:s27652), importin-13 (ID:s18609), RanBP6 (ID:s25641), RanBP9 (ID:s19529), exportin-7 (RanBP16, ID:s22894), exportin-2 (CSE1L, ID:s3588), exportin-4 (ID:s34638), exportin-5 (ID:s33190), exportin-6 (ID: s23302), exportin-T (ID:s22232), RanBP17 (ID:s35075), Hikeshi (ID:s28222), Calreticulin (ID:s114).

### Expression and purification of recombinant proteins

GST-fusion proteins were expressed in *Escherichia coli* strain BL21 and RosettaBlue (Merck Millipore), and purified with Glutathione Sepharose 4B (17075601, Cytiva). GST cleavage was performed using Turbo3C Protease (Accelagen, San Diego, CA, USA). Hexahistidine (6 × His)-RanQ69L was expressed in BL21 and purified with TALON Metal Affinity Resin (Takara Bio Inc.). The GTP-bound RanQ69L was purified as described previously^64^.

### GST pulldown assay

At 24 h after transfection, HeLa cells were washed with cold PBS. The cells were lysed with lysis buffer (20 mM HEPES/NaOH [pH 7.3], 150 mM NaCl, 5 mM MgCl_2_, 0.05% Tween-20, 1 μg/mL each of aprotinin, leupeptin, and pepstatin A). These lysates were centrifuged at 20,400×*g* for 30 min, and the supernatants were collected and quantified using a DC Protein Assay kit (Bio-Rad Laboratories, Inc., Hercules, CA, USA). GST pulldown assays were performed by mixing cell lysates with Glutathione Sepharose 4B charged with 50 pmol GST fusion proteins with or without 2 µM 6 × His-RanQ69L. The beads were washed with lysis buffer and resuspended in Laemmli sample buffer, and subsequently analysed by SDS-PAGE and western blotting. Quantification of western blot band signals was performed with CS Analyzer software and statistical analysis was performed using GraphPad Prism.

### Immunofluorescence

HeLa cells and MCF-7 cells grown on glass cover slides in a 12-well culture plates were fixed with formaldehyde for 10 min at room temperature, permeabilized with PBS containing 0.2% TritonX-100 and washed with PBS. After blocking with blocking buffer (137 mM NaCl, 2.7 mM KCl, Na_2_HPO_4_, KH_2_PO_4_, 1% BSA, 2% horse serum, 0.1% gelatin, 0.1% Triton X-100, 0.05% Tween 20, 0.05% sodium azide), the cells were incubated with primary antibodies. After washing with PBS, Alexa Fluor Plus 488- and 555-conjugated secondary antibodies (Thermo Fisher Scientific) were used. Nuclei were stained with 0.1 μg/mL DAPI (Dojindo, Kumamoto, Japan). Fluorescence was observed using an Olympus FV-1200 confocal microscope (Olympus, Tokyo, Japan). Quantification of nuclear signal was performed with ImageJ software, and statistical analysis was performed in GraphPad Prism.

### Quantitative RT-PCR

Real-time PCR reactions were carried out using an ABI PRISM 7900 (Thermo Fisher Scientific). The amplicons were designed to amplify fragments of > 150-bp (Supplementary Table 2). A One Step SYBR PrimeScript RT-PCR Kit II (Takara Bio Inc.) was used for the one-step RT-PCR reactions containing total RNA from siRNA-transfected HeLa cells as a template. According to the manufacturer’s protocol, reverse transcription was conducted at 42 °C for 5 min and then 95 °C for 10 s, followed by an initial activation at 95 °C for 5 s and 60 °C for 30 s for a total of 40 cycles.

## Supplementary information

Supplementary Information.

## Data Availability

The datasets generated and/or analysed during the current study are available from the corresponding author upon reasonable request.
