# Supplementary material for: Transportin-2 plays a critical role in nucleocytoplasmic shuttling of oestrogen receptor-α
Source: Sci Rep. 2020 Oct 29;10:18640. doi: 10.1038/s41598-020-75631-3 (PMC7596556; doi:10.1038/s41598-020-75631-3)
Supplement: Supplementary file 1 — Supplementary Information. [file 41598_2020_75631_MOESM1_ESM.pdf]

Supporting information

Transportin-2 plays a critical role in nucleocytoplasmic shuttling of oestrogen receptor-α

Tetsuji Moriyama[1], Yoshihiro Yoneda[2,3], Masahiro Oka[3,4] and Masami Yamada[1,5*].

1. Department of Cell Biology and Biochemistry, Division of Medicine, Faculty of Medical Sciences, University of Fukui, 23-3 Matsuoka Shimoaizuki, Eiheiji-cho, Yoshida-gun, Fukui, 910-1193, Japan
2. National Institutes of Biomedical Innovation, Health and Nutrition (NIBIOHN), 7-6-8 Saito-Asagi, Ibaraki, Osaka 567-0085, Japan
3. Laboratory of Nuclear Transport Dynamics, Graduate School of Pharmaceutical Sciences, Osaka University, 1-6 Yamada-oka, Suita, Osaka 565-0871
4. Laboratory of Nuclear Transport Dynamics, National Institutes of Biomedical Innovation, Health and Nutrition (NIBIOHN), 7-6-8 Saito-Asagi, Ibaraki, Osaka 567-0085, Japan
5. Life Science Research Laboratory, University of Fukui, 23-3 Matsuoka Shimoaizuki, Eiheiji-cho, Yoshida-gun, Fukui, 910-1193, Japan

Corresponding authors:
Department of Cell Biology and Biochemistry, Division of Medicine, Faculty of Medical Sciences, University of Fukui, 23-3 Matsuoka Shimoaizuki, Eiheiji-cho, Yoshida-gun, Fukui, 910-1193, Japan. Tel.: 81-776-61-8315; E-mail: yamadama@u-fukui.ac.jp

Supplementary Figure 1-13
Supplementary Table 1 and 2

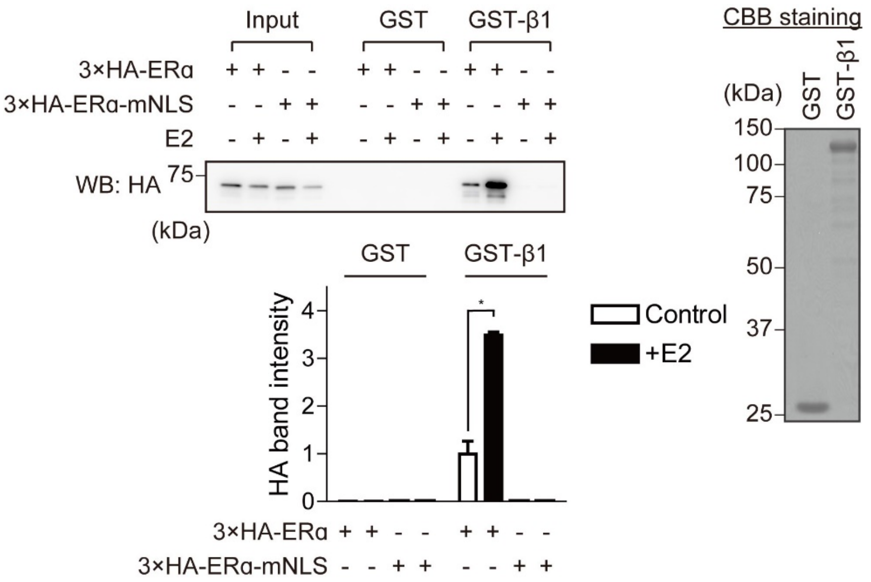

Supplementary Figure 1. Detection of ERα-importin-β1 binding by GST-pulldown assay. GST-pulldown assays were performed using recombinant GST alone or GST-importin-β1 (GST-β1) with HeLa lysates expressing 3×HA-ERα or 3×HA-ERα-mNLS, in the absence or presence of 10 nM E2. CBB staining provided the loading control for the reaction. The average band intensity of 3×HA-ERα bound to GST-importin-β1 in the absence of E2 was set to 1. Each data point represents the average of data from three independent experiments and the error bars denote the standard deviation. *$P < 0.05$, the Paired ratio t-test, compared with the control.

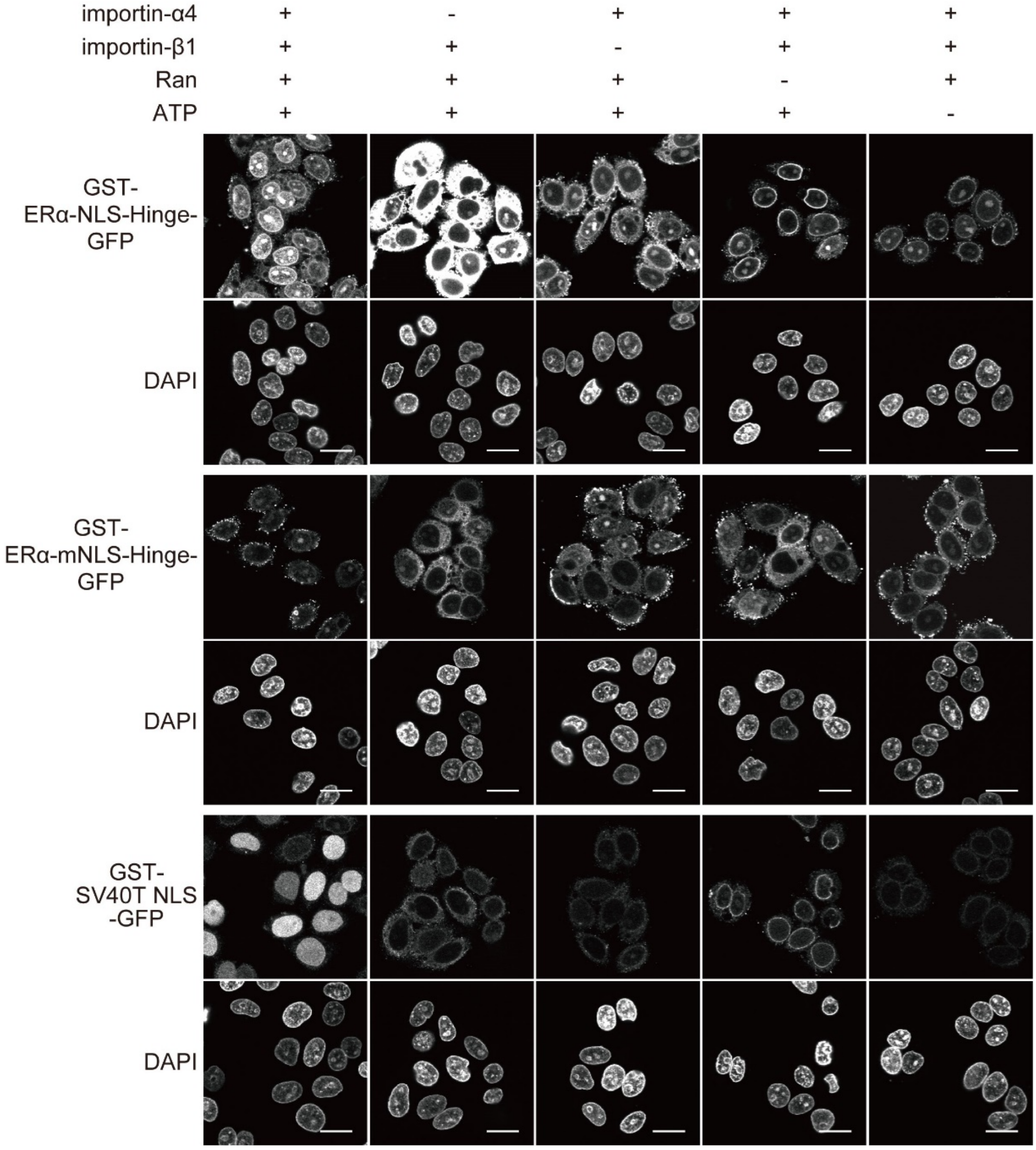

Supplementary Figure 2. Analysis of the ERα nuclear import pathway depends on importin-α4. An *in vitro* transport assay was performed to test the nuclear import of GFP conjugated GST-ERα-NLS-Hinge, or GST-ERα-mNLS-Hinge, with or without importin-α4, importin-β1, RanGDP, or an ATP-regenerating system containing GTP. GST-SV40T NLS-GFP was used as the positive control. Scale bars, 20 μm.

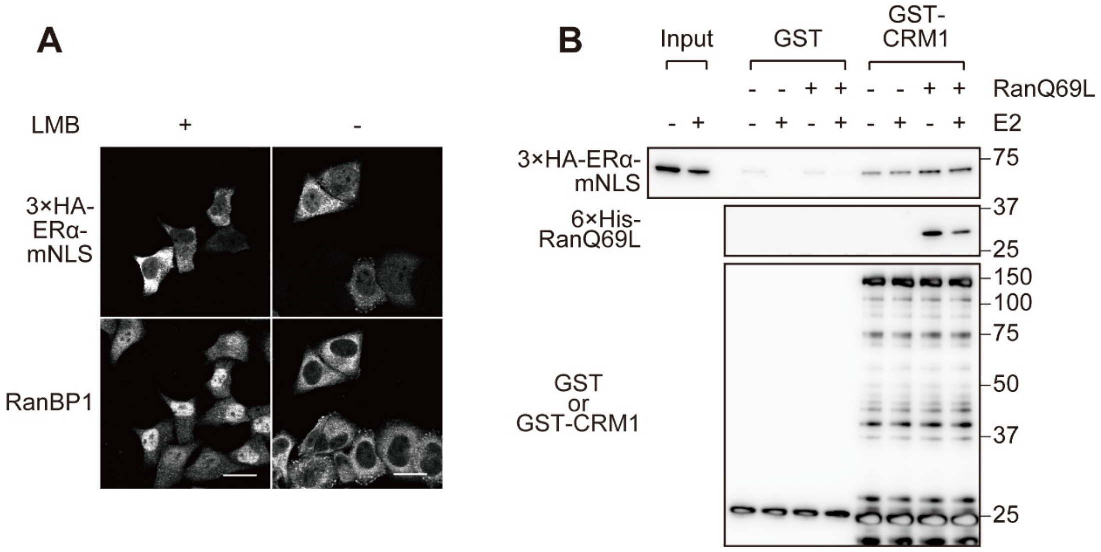

Supplementary Figure 3. Analysis of the nuclear export of ERα-mNLS (**A**) The effects of leptomycin B (LMB), a specific inhibitor of CRM1-dependent nuclear export, on the subcellular distribution of 3×HA-ERα-mNLS and RanBP1 in HeLa cells. The cells were treated with or without 10 nM LMB in the presence of E2 for 3 h. LMB inhibited the nuclear export of endogenous RanBP1, which was used as the positive control. Scale bars, 20 µm. (**B**) CRM1 recognises 3×HA-ERα-mNLS in a RanGTP-sensitive manner. GST-pulldown assays were performed with recombinant GST or GST-CRM1 using HeLa lysates expressing 3×HA-ERα-mNLS, in the presence or absence of 2 µM recombinant 6×His-RanQ69L.

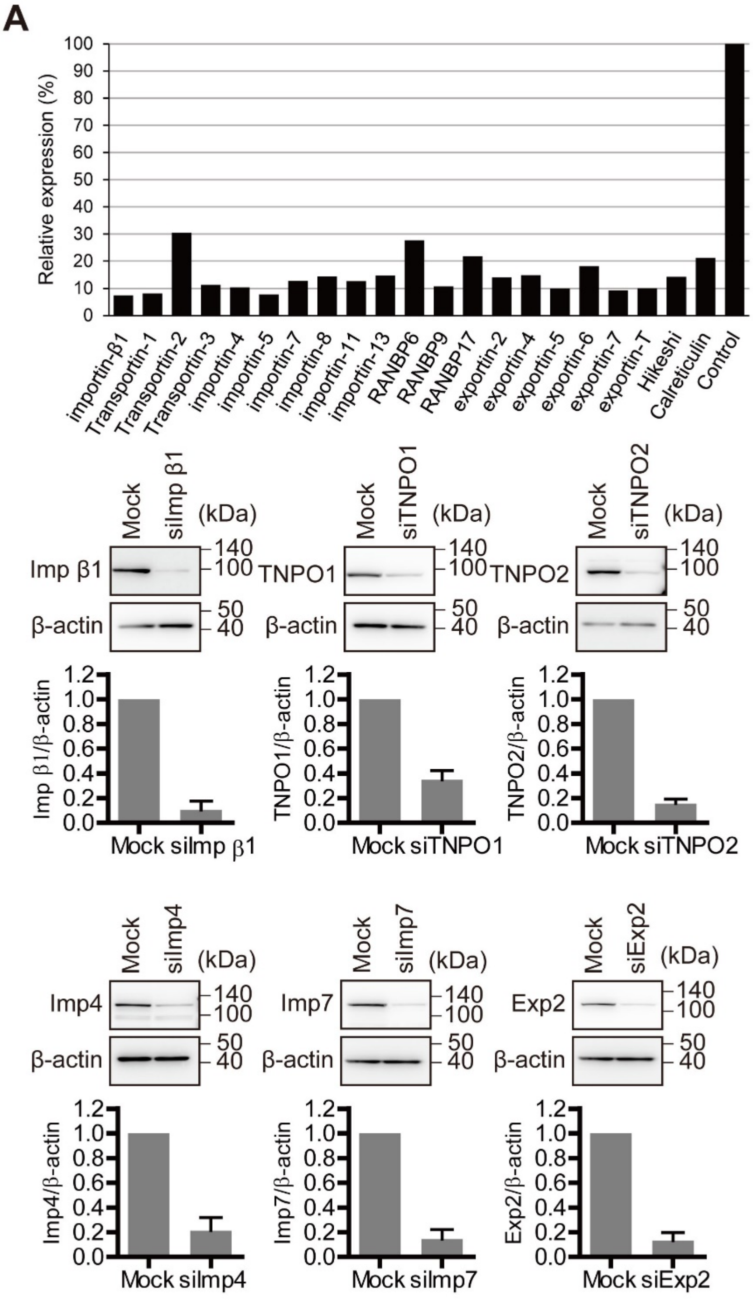

Supplementary Figure 4. The efficiency of RNAi-mediated knockdown was assessed by quantitative real-time PCR and western blotting. (**A**) Real-time PCR was performed using gene-specific primers for importin-βs, hikeshi, and calreticulin, and the total RNA isolated from the siRNA-transfected HeLa cells was used as the template. The data are expressed as the average copies of each mRNA divided by the mRNA level of the housekeeping gene, hypoxanthine phosphoribosyltransferase (HPRT), and compared with that of the control cells, which was set to 100%. (**B**) Importin-β protein levels were measured by western blotting in lysates of HeLa cells transfected with siRNA against

importin-β1 (Imp β1), transportin-1 (TNPO1), transportin-2 (TNPO2), importin-4 (Imp4), importin-7 (Imp7) or exportin-2 (Exp2). The importin-β/β-actin ratio of the mock sample was set to 1. Each data point represents the average of three independent experiments and the error bars denote the standard deviation.

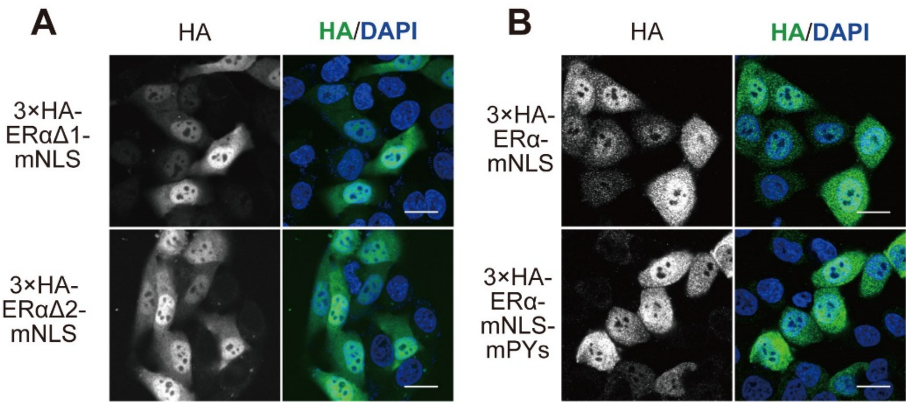

Supplementary Figure 5. Immunofluorescence images of (**A**) 3×HA-ERαΔ1-mNLS (61-595 residues), or 3×HA-ERαΔ2-mNLS (181-595 residues), (**B**) 3×HA-ERα-mNLS, or 3×HA-ERα-mNLS-mPYs in HeLa cells, in the absence of E2 for 3 h. The cells were immunostained for HA (green) and stained with DAPI (blue). Scale bars, 20 µm.

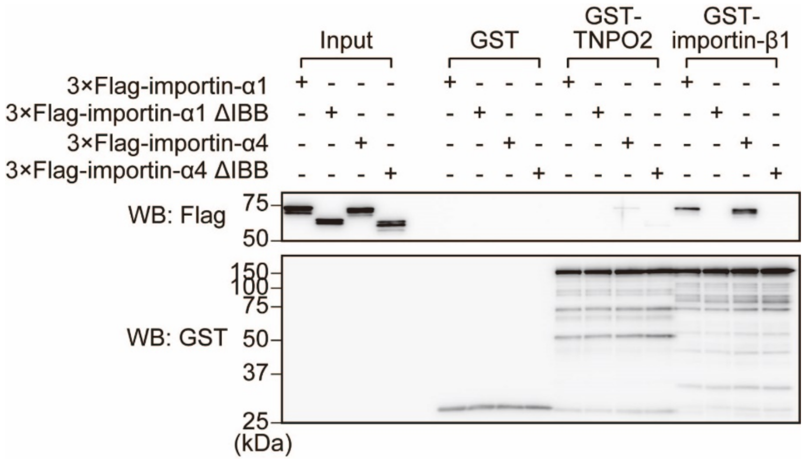

Supplementary Figure 6. Detection of importin-α-TNPO2 binding by GST-pulldown assays. The assays were performed with recombinant GST, GST-TNPO2, or GST-importin-β1 (positive control) using recombinant 3×Flag-importin-α1, 3×Flag-importin-α1 lacking the N-terminal importin-β1 binding domain (ΔIBB), 3×Flag-importin-α4, or 3×Flag-importin-α4 ΔIBB. The bound proteins were analysed by western blotting using an anti-Flag antibody.

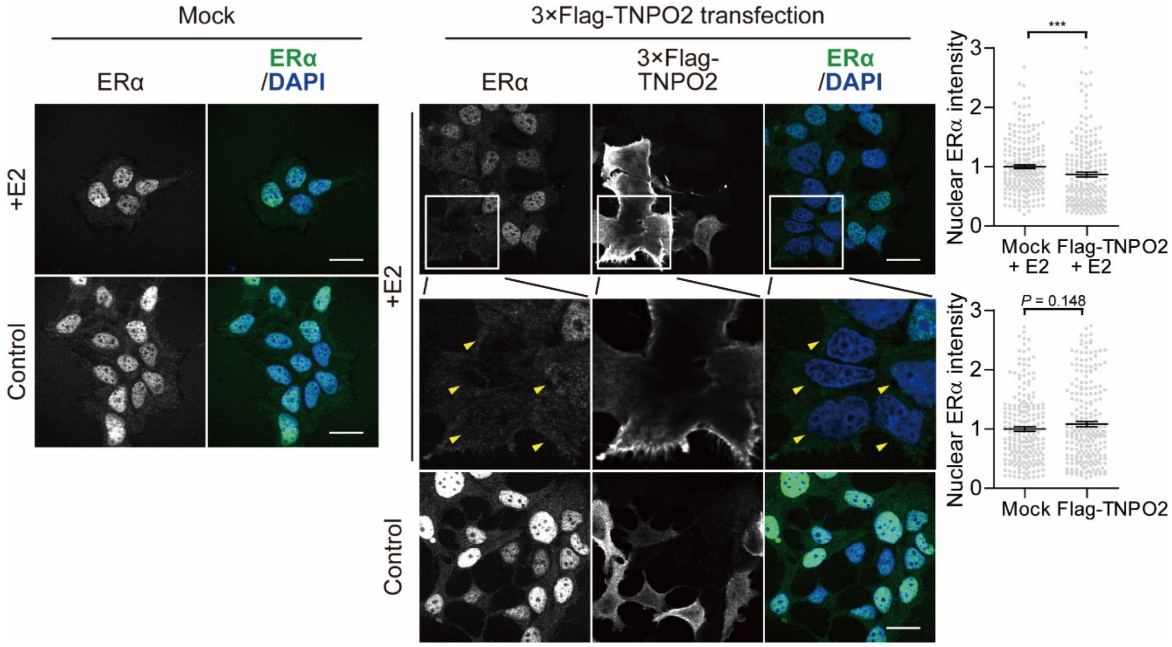

Supplementary Figure 7. Immunofluorescence images of MCF-7 cells transfected with plasmids expressing 3×Flag-TNPO2, in the presence or absence of 10 nM E2, for 10 min. The cells were immunostained for ERα (green) and Flag, and stained with DAPI (blue). The overexpression of 3×Flag-TNPO2 (yellow arrow) induced a noticeable change in the translocation of the endogenous ERα from the nucleus to the cytoplasm following treatment with E2. Scale bars, 20 µm. The graphs depict the intranuclear ERα staining intensity calculated from the average of at least 200 cells, and the error bars denote the corresponding standard error. $^{***}P < 0.001$ determined by the Mann-Whitney test through comparison with the mock samples, in the presence or absence of E2.

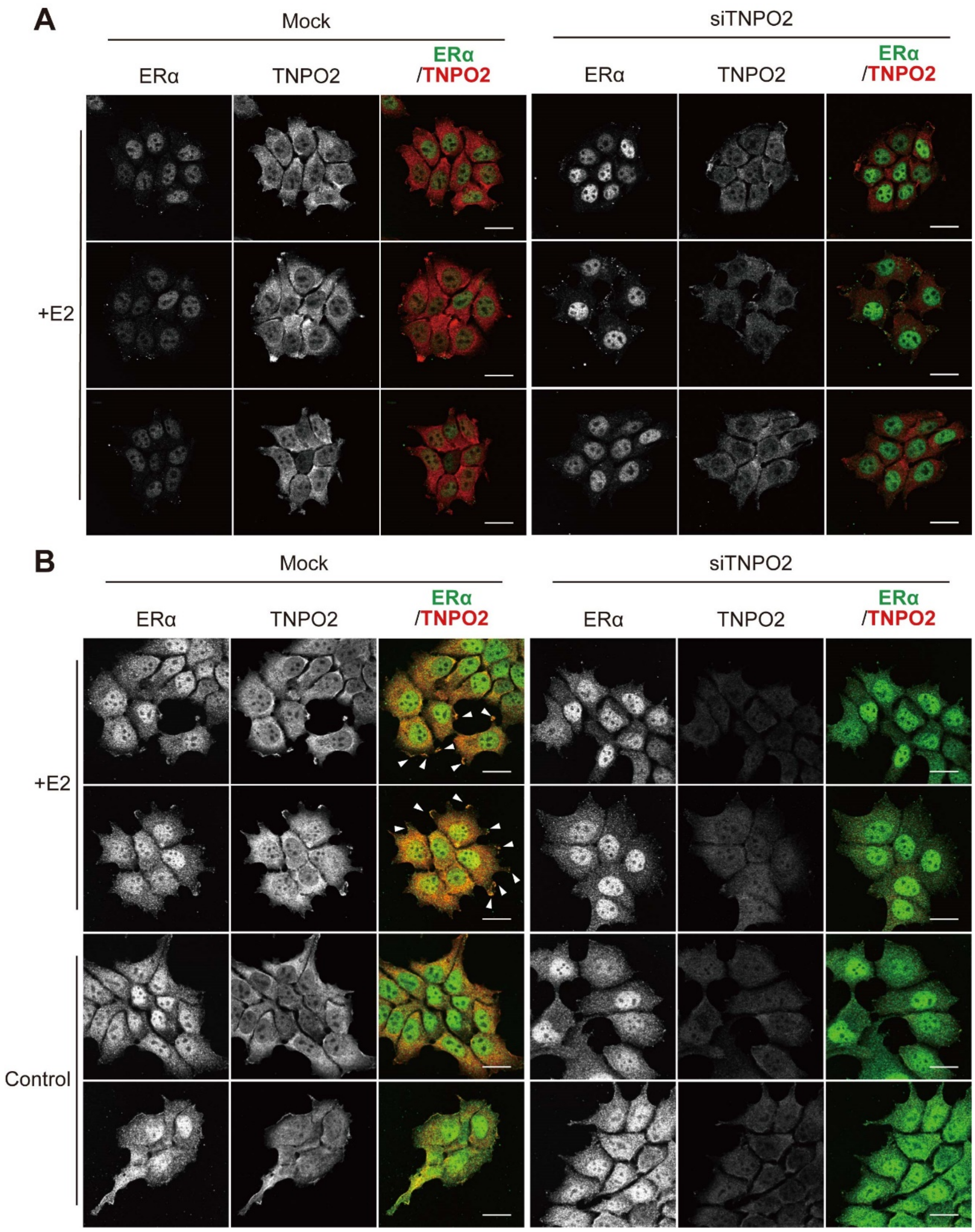

Supplementary Figure 8. Immunofluorescence images of the endogenous ERα and TNPO2 in MCF-7 cells transfected with TNPO2-specific siRNAs in the presence of 10 nM E2, for 10 min. (**A**) The cells were immunostained for ERα (green) and TNPO2 (red). All the images were captured under the same settings. Images in (**B**) show the enhanced fluorescence signals of Can Get Signal Immunostain A solution (NKB-501,

TOYOBO, Osaka, Japan) for detecting the ERα located on the cell membrane. The endogenous TNPO2 localised on the plasma membrane and colocalised with ERα (arrow). Scale bars, 20 μm.

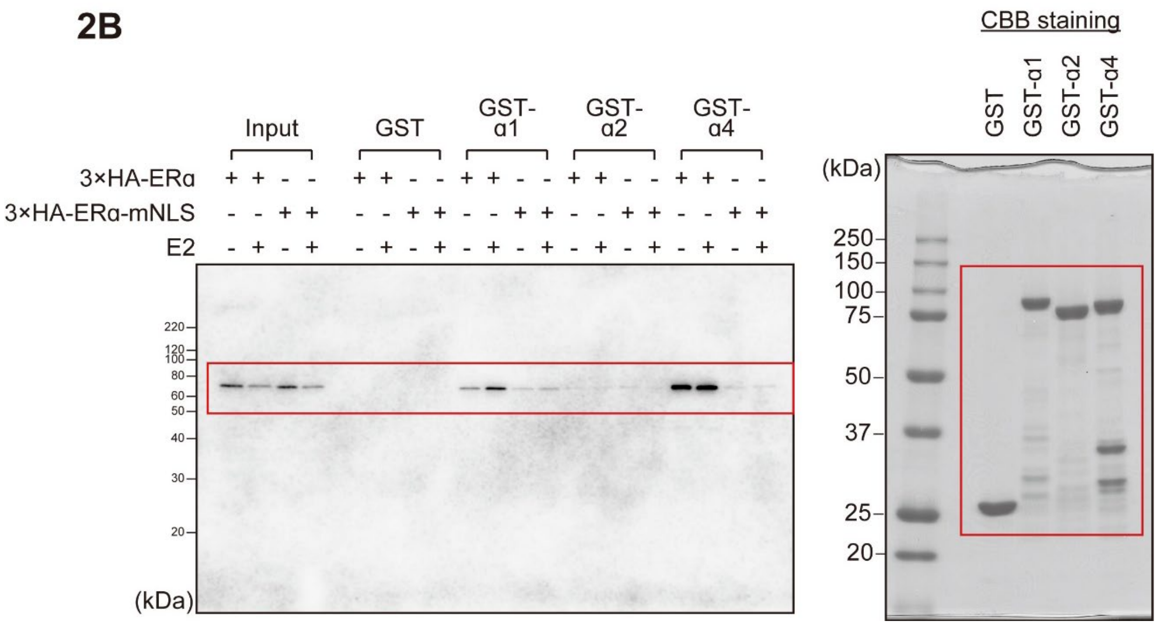

Supplementary Figure 9. Full-length images of western blot and CBB staining in Fig. 2B. Importin-α recognises the basic amino acids of ERα. GST pulldown assays were performed with recombinant GST, GST-importin-α1 (GST-α1), GST-importin-α2 (GST-α2) and GST-importin-α4 (GST-α4), using HeLa lysates expressing 3×HA-ERα or 3×HA-ERα-mNLS, in the presence or absence of 10 nM E2. CBB stain was used as the loading control for the reaction.

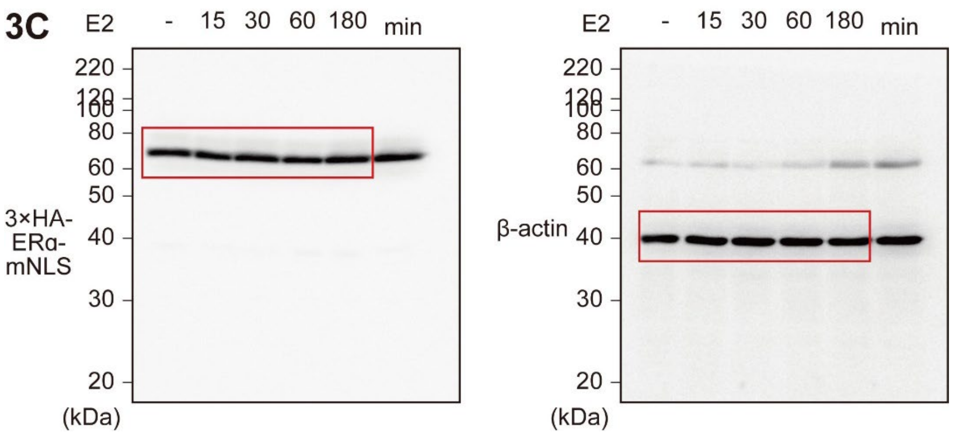

Supplementary Figure 10. Full-length images of western blot in Fig. 3C. Treatment with E2 did not affect the steady-state expression levels of 3×HA-ERα-mNLS. HeLa cells expressing 3×HA-ERα-mNLS were treated with 10 nM E2 for the indicated durations. Following treatment, the cells were lysed and subjected to SDS-PAGE. Western blotting was performed with an anti-HA antibody. β-actin was used as the loading control.

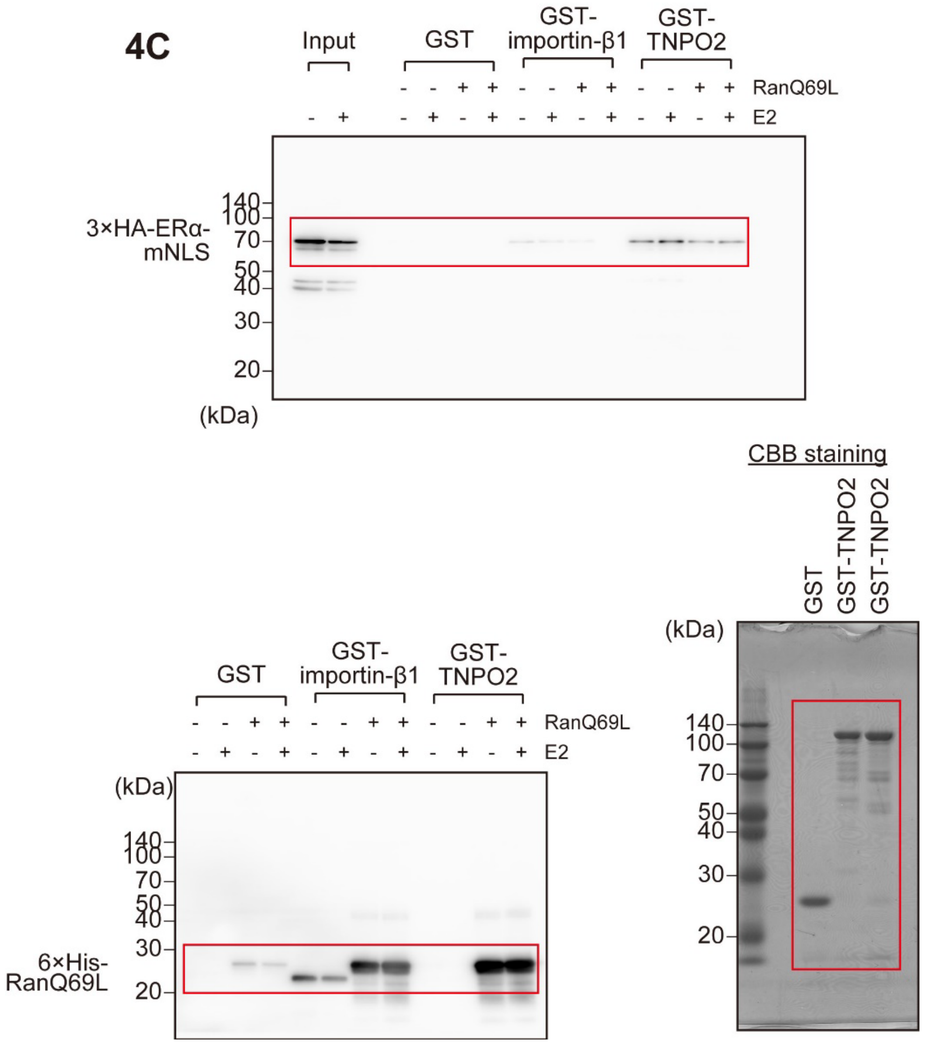

Supplementary Figure 11. Full-length images of western blot and CBB staining in Fig. 4C. TNPO2 recognises 3×HA-ERα-mNLS in an E2-dependent and RanGTP-sensitive manner. GST pulldown assays were performed with recombinant GST, GST-importin-β1 and GST-TNPO2, using HeLa lysates expressing 3×HA-ERα-mNLS, in the presence or absence of 2 μM recombinant 6×His-RanQ69L. GST-importin-β1 was used as the negative control.

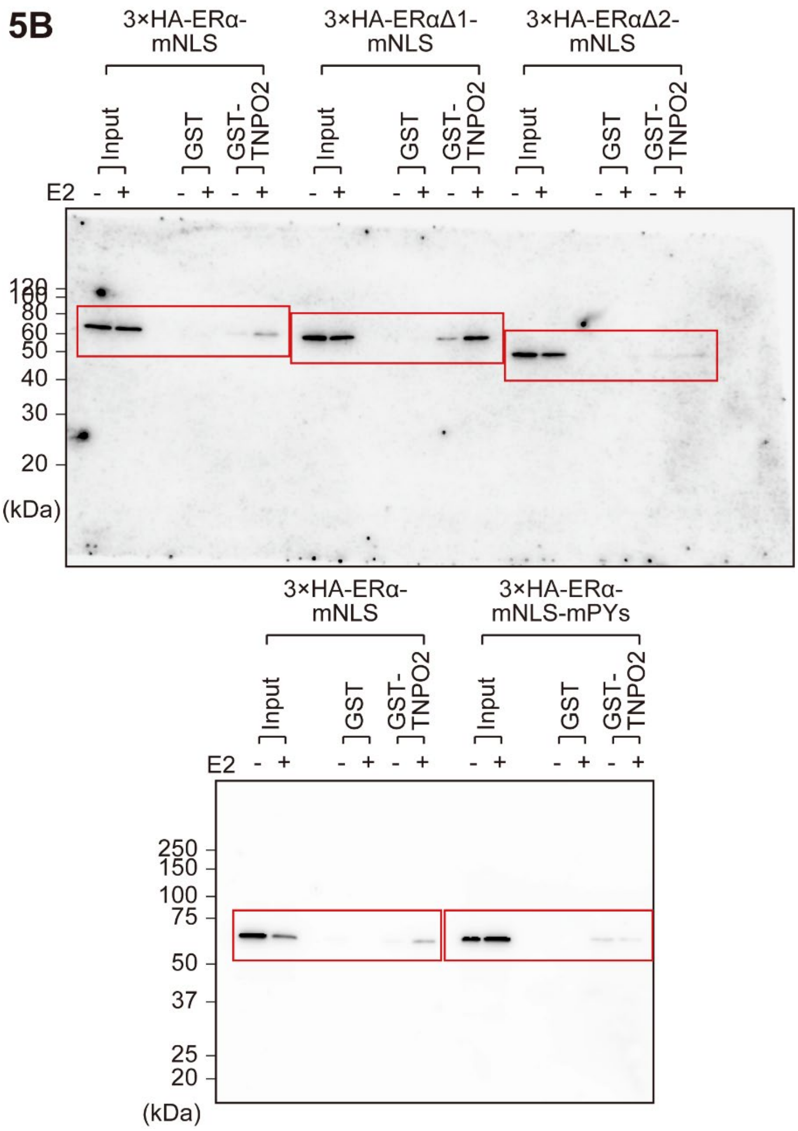

Supplementary Figure 12. Full-length images of western blot in Fig. 5B. GST pulldown assays were performed with recombinant GST and GST-TNPO2, using HeLa lysates expressing 3×HA-ERα-mNLS, 3×HA-ERαΔ1-mNLS, or 3×HA-ERαΔ2-mNLS, and 3×HA-ERα-mNLS or 3×HA-ERα-mNLS-mPYs

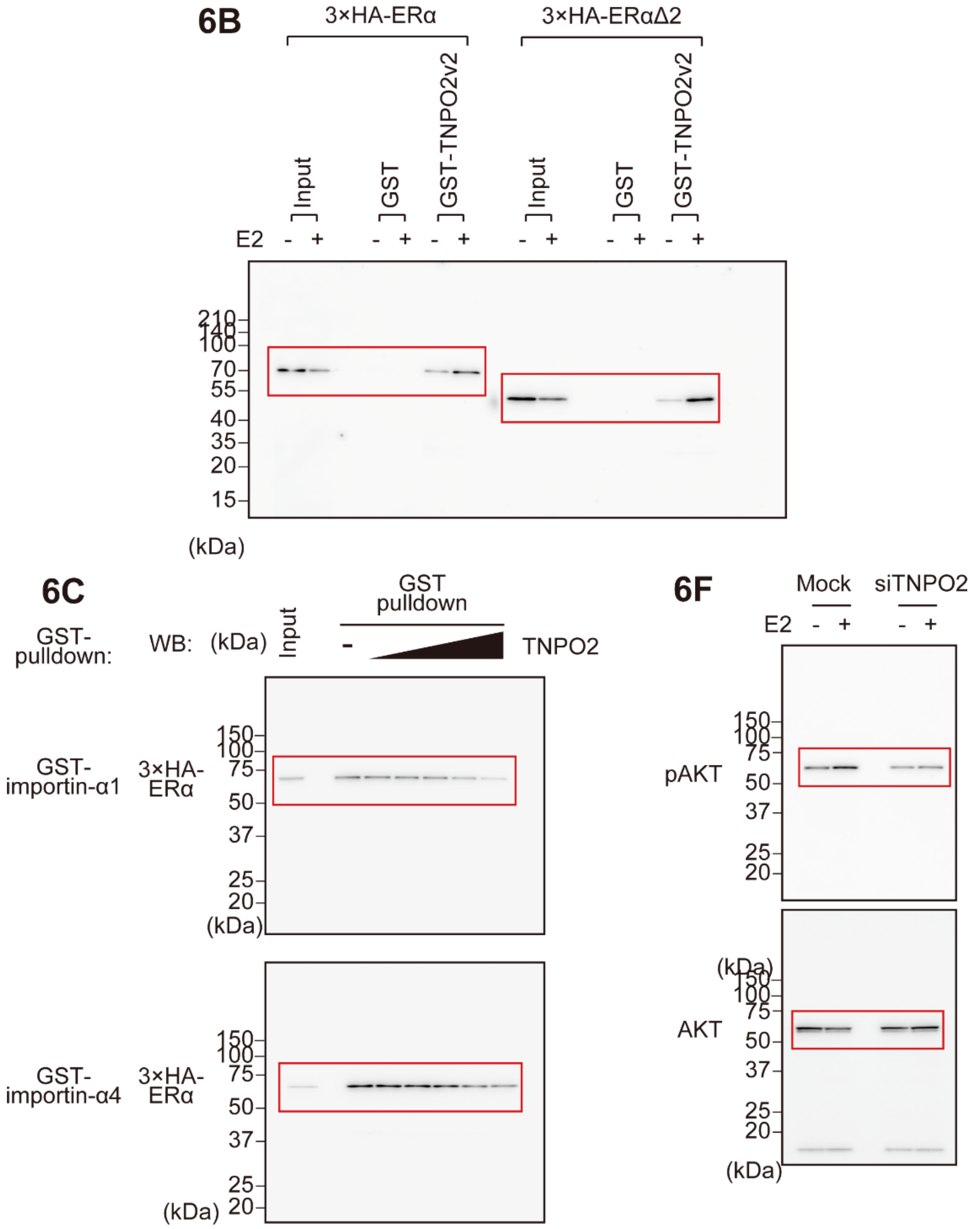

Supplementary Figure 13. Full-length images of western blot in Fig. 6. (**B**) GST pulldown assays were performed with recombinant GST and GST-TNPO2, using HeLa lysates expressing 3×HA-ERα or 3×HA-ERαΔ2. (**C**) Competitive binding of importin-α1 or importin-α4 and TNPO2 to ERα. GST-importin-α1 or GST-importin-α4 were immobilised on glutathione Sepharose beads and incubated with HeLa lysates expressing 3×HA-ERα, in the presence of 10 nM E2. The beads were washed and incubated with different amounts of TNPO2. The bound fraction was analysed by immunoblotting for HA. (**F**) TNPO2 knockdown inhibited the E2-dependent AKT activation. MCF-7 cells were transfected with TNPO2-specific siRNAs, and treated

with 10 nM E2 for 15 min. The cells were lysed and subjected to SDS-PAGE. Western blotting was performed using anti-phosphorylated-AKT and anti-AKT antibodies.

Name	Sequence
TNPO2 Fw	ACCgccgccaccATGGACTGGCAGCCAGACGA
TNPO2 Re	GGCGCGCCCACCCTTCTAGACCCCATAGAAAGCCGCCA
importin-β1 Fw	ACCgccgccaccATGGAGCTGATCACCATTCTCGAGAAGA
importin-β1 Re	GGCGCGCCCACCCTTTCAAGCTTGGTTCTTCAGTTTCCTC
importin-α1 Fw	accgccgccaccATGACCACCCCAGGAAAAGA
importin-α1 ΔIBB Fw	accgccgccaccGAAGAAGAAGTTATGTCAGATGGAG
importin-α1 Re	ggcgcgcccacccttTCAAAGCTGGAAACCTTCCA
importin-α2 Fw	ACCgccgccaccATGTCCACCAACGAGAATGCTAATACAC
importin-α2 Re	GGCGCGCCCACCCTTCTAAAAGTTAAAGGTCCCAGGAGCC
importin-α4 Fw	accgccgccaccATGGCGGACAACGAGAAA
importin-α4 ΔIBB Fw	accgccgccaccGACTCTGATATAGATGGTGATTATA
importin-α4 Re	ggcgcgcccacccttCTAAAACTGGAACCCTTCTGT
ERα Fw	CGAAGCTTGGGATTCATGACCATGACCCTCCACACCAAAGCATCT
ERα Re	GGCGCGCCCACCCTTTCAGACCGTGGCAGGGAAACCCTCT
ERα-m1 Fw	GCTGCTTGCTACGAAGTGGGAATGATGAAAGGTGGGATA
ERα-m1 Re	GAGCCGGCAGGCCTGGCAGCTCTTC
ERα-m2 Fw	GCTGCTGACGCTGCTGGAGGGAGAATGTTGAAACACAAGC
ERα-m2 Re	TATCCCACCTTTCATCATTCCCACTTC
ERα-m3 Fw	CAGAGAGATGATGGGGAGGGCAGGG
ERα-m3 Re	AGCAGCGTGAGCCAACATTCTCCCTCC
ERα-mPY1 Fw	gCCgcCGGCCCCGGGTCTGAGGCTGCGGCGTTC
ERα-mPY1 Re	GAGGCCGGTCTGACCGTAGACCT
ERα-mPY2 Fw	gCCgcCTACCTGGAGAACGAGCCCAGCGGCTACA
ERα-mPY2 Re	CACCTGCTGGCCGTGGGGCTGCA
ERα-61- Fw	GAGTTCAACGCCGCGGCC
ERα-181- Fw	GAGACTCGCTACTGTGCAGTGTGCAATG
ERα-243- Fw	ataGAATTCCGTAAATGCTACGAAGTGGGA
ERα-mNLS-243- Fw	ataGAATTCGCTGCTTGCTACGAAGTGGGA
ERα-302- Re	ataGGTACCTCTTAGAGCGTTTGATCATGA
Bgl2-M9 peptide Fw	accagatctAATCAGTCTTCAAATTTTGGACCCATGA
Xho1-M9 peptide Re	aaactcgagATAGCCACCTTGGTTTCGTGGT

Supplementary Table 1. Primers used for plasmid construction

Name	Sequence
importin-β1 Fw	aatggatttggccattgaag
importin-β1 Re	tcgcataaaacttgctggtg
Transportin-1 Fw	tgatgatacaatttctgactggaatc
Transportin-1 Re	ggcagcagttcatcacgata
Transportin-2 Fw	acattgctgttccagtgcat
Transportin-2 Re	tgaagcaggctttggtgag
Transportin-3 Fw	gatcccagcggaaaggag
Transportin-3 Re	agatctcccatgcatgaacc
importin-4 Fw	ctctgtggcgtgctcaag
importin-4 Re	caacatggcgtcgtattca
importin-5 Fw	tgctctagatcgaatggcttg
importin-5 Re	atgccggtatttccagtcag
importin-7 Fw	gtgaacagggatgtacctaatgaa
importin-7 Re	atgtaaggcccacttcttgc
importin-8 Fw	gctgtggcaagaagatccat
importin-8 Re	aagagagtctgggctgctgt
importin-11 Fw	gaagaagacccagaaggcttta
importin-11 Re	cagtgcatggcctcaaacta
importin-13 Fw	tcacctccgtcacactcact
importin-13 Re	gcataaatgaatcaacaatatcagga
RanBP6 Fw	aaaaactttgtgatatttttgcagtg
RanBP6 Re	gccagtggttagtgccatc
RanBP9 Fw	gattgtgacaccgaaatgga
RanBP9 Re	caaagtggatcattctttctatgg
RanBP17 Fw	atgcagcaaaacccagatgt
RanBP17 Re	ccactggttccgacagtctt
exportin-2 Fw	tgtaaacctaactgagttctttgtgaa
exportin-2 Re	ccgtcagctttaaggacagg
exportin-4 Fw	gttaccttgcactcgccaat
exportin-4 Re	catagctatataatgtctgcccaaat
exportin-5 Fw	tatatactctccgccgcaca
exportin-5 Re	gcctcttcagaaagacgtagtgt
exportin-6 Fw	aggtggtggccaaagtgat
exportin-6 Re	ttgtcctgaagaacagggaac
exportin-7 Fw	ccatggtcctaagcgtcttc
exportin-7 Re	aacggccccagtacttcaa
exportin-T Fw	gggtttttcagcattgacca
exportin-T Re	tgtcccattccatttaccaac

Name	Sequence
Hikeshi Fw	ttagtctcgactagggcagtagc
Hikeshi Re	tgctgtgcagctgtttgc
Calreticulin Fw	ctatgataactttggcgtgctg
Calreticulin Re	actcctcagcgtatgcctca
HPRT Fw	tgaccttgatttattttgcatacc
HPRT Re	cgagcaagacgttcagtcct

Supplementary Table 2. Primers used for Real-time PCR assay
